# Association between moderated level of air pollution and fetal growth: the potential role of noise exposure

**DOI:** 10.1038/s41598-021-90788-1

**Published:** 2021-05-27

**Authors:** Anne-Sophie Mariet, Nadine Bernard, Sophie Pujol, Paul Sagot, Gérard Thiriez, Didier Riethmuller, Mathieu Boilleaut, Jérôme Defrance, Hélène Houot, Anne-Laure Parmentier, Eric Benzenine, Frédéric Mauny, Catherine Quantin

**Affiliations:** 1 Service de Biostatistiques et d’Information Médicale, CHU Dijon Bourgogne, 21000 Dijon, France; 2 Inserm, Clinical Investigation Center of Dijon (Inserm CIC 1432), CHU Dijon Bourgogne, 21000 Dijon, France; 3 Inserm, Biostatistique, Biomathématique, Pharmacoépidémiologie et Maladies Infectieuses (B2PHI), UMR 1181, Université Bourgogne Franche-Comté, 21000 Dijon, France; 4 Laboratoire Chrono-Environnement UMR 6249, CNRS, Université de Bourgogne Franche-Comté, 25000 Besançon, France; 5 Laboratoire ThéMA UMR 6049, CNRS, Université de Bourgogne Franche-Comté, 25000 Besançon, France; 6 Unité de Méthodologie en Recherche Clinique, épidémiologie et santé publique, INSERM CIC 1431, CHU de Besançon, 25000 Besançon, France; 7 Service de Gynécologie-Obstétrique, CHU Dijon Bourgogne, 21000 Dijon, France; 8 Service de Réanimation Pédiatrique, Néonatalogie et Urgences Pédiatriques, CHU de Besançon, 25000 Besançon, France; 9 Service de Gynécologie-Obstétrique, CHU de Besançon, 25000 Besançon, France; 10 Atmo Bourgogne-Franche-Comté, 25000 Besançon, France; 11 Centre Scientifique et Technique du Bâtiment, Pôle Acoustique et Eclairage, 38400 Saint Martin d’Hères, France

**Keywords:** Environmental impact, Epidemiology

## Abstract

This study aims to analyze, in a population of singletons, the potential confounding or modifying effect of noise on the relationship between fetal growth restriction (FGR) or small for gestational age (SGA) and environmental exposure to air pollution. All women with single pregnancies living in one of two medium-sized cities (Besançon, Dijon) and who delivered at a university hospital between 2005 and 2009 were included. FGR and SGA were obtained from medical records. Outdoor residential exposure to nitrogen dioxide (NO_2_) and particulate matter (PM_10_) was quantified at the mother’s address at delivery over defined pregnancy periods; outdoor noise exposure was considered to be the annual average daily noise levels in the façade of building (L_Aeq,24 h_). Adjusted odds ratios (OR_a_) were estimated by multivariable logistic regressions. Among the 8994 included pregnancies, 587 presented FGR and 918 presented SGA. In the two-exposure models, for SGA, the OR_a_ for a 10-µg/m^3^ increase of PM_10_ during the two last months before delivery was 1.18, 95%CI 1.00–1.41 and for FGR, these OR_a_ were for the first and the third trimesters, and the two last months before delivery: 0.77 (0.61–0.97), 1.38 (1.12–1.70), and 1.35 (1.11–1.66), respectively. Noise was not associated with SGA or FGR and did not confound the relationship between air pollution and SGA or FGR. These results are in favor of an association between PM_10_ exposure and fetal growth, independent of noise, particularly towards the end of pregnancy, and of a lack of association between noise and fetal growth.

## Introduction

The question of the influence of environmental conditions during pregnancy on adverse pregnancies outcomes is increasingly studied but remains debated. Among the adverse outcomes of pregnancy, fetal growth abnormalities are associated with poor perinatal outcomes^1–6^ and increase the prevalence of long-term neurodevelopmental, cardiovascular, and endocrine consequences^6–9^.

Among urban environmental exposures, air pollution and noise are the most ubiquitous and relatively correlated^10^.

The exposure to noise induces the release of stress hormones and inflammatory signaling molecules leading to oxidative stress and vascular dysfunction, and the exposure to air pollution has been suspected of increasing oxidative stress and systemic inflammation, and, during pregnancy, it may decrease uterine blood flow and placental fetal exchange, therefore slowing fetal growth^8,11–20^.

The association between air pollution and fetal growth disorders has been identified in many of studies in various more and less exposed regions of the world, with different pollutants and different fetal growth indicators measured in newborns at term or not: birth weight (BW), low birth weight (LBW), small for gestational age (SGA), or ultrasound antenatal measurements^21–25^. Nieuwenhuijsen's review in 2017 identified studies of the effects of noise on adverse pregnancy outcomes including fetal growth^18^. The authors found low quality evidence for an association between road traffic noise and LBW and SGA. They conclude that good quality studies were needed in various regions, particularly at lower levels of noise, and taking into account confounding factors like air pollution. Indeed, only a few studies have taken into account the effect of the exposure to both air pollution and noise^26–32^. The last two studies were published after the Nieuwenhuijsen's review^31,32^. In London, Smith et al. estimated that 3% of infants born at term with LBW are directly attributable to residential exposure to PM_2.5_ > 13.8 μg/m^3^ during pregnancy, and their results suggested little evidence of an independent exposure–response effect of traffic related noise (daytime and night-time road traffic noise) on BW at term^32^. In 2019, Nieuwenhuijsen et al. studied the influence of the urban exposome on BW in six European birth cohorts and found no evidence of an association between road traffic noise or air pollution and BW and LBW at term in co-exposure analyses^31^.

Although the adverse effect of air pollution on fetal growth is generally recognized, the influence of noise remains debated, as does the relative contribution of these two types of pollutants and the potential effects of co-exposure on fetal growth. The objective of this study was to explore the potential confounding or modified effect of noise on the relationship between fetal growth and environmental exposure to moderate levels of air pollution.

## Material and methods

### Population

For this retrospective study, we included all single pregnancies in women residing in Besançon or in the urban area of Dijon who delivered at the Besançon or Dijon university hospital between 1st January 2005 and 31st December 2009. The Besançon and Dijon university hospitals are level 3 maternities (i.e. with a neonatal intensive care unit). Stillborns and live newborns, whose births occurred after 22 completed weeks of gestation or with birth weight > 500 g, were included. When a woman had several single pregnancies in the study period, only one pregnancy was included after random selection. Hence, the number of women and the number of pregnancies included in the analysis population is the same (N = 8994).

This work is part of the PRECEE program (PREgnancy and Combined Environmental Exposure) and complements results published by Mariet et al. in 2018 on multiple pregnancies, by Barba-Vasseur et al. in 2017 on preterm birth in single pregnancy, and by Brembilla et al. in 2019 on vulnerability markers in pregnant women^33–35^.

### Outcomes

Birth weight and fetal growth restriction (FGR) were extracted from the Besançon computerized medical records (DIAMM software (version 7.5, http://www.micro6.fr/mere-enfant.php) developed by the Association of Computerized Users in Pediatrics, Obstetrics and Gynecology^36^) and from the Burgundy perinatal network records and paper medical records for Dijon. Births were classified as SGA if birth weight was < 10th centile for gestational age (in weeks) and sex. The threshold for the 10th centile of birth weight was estimated in a population of French newborns from single and multiple pregnancies by gestational age and sex^36^. In order to test for a classification effect, SGA was also defined according to another birth weight standard for gestational age and sex, estimated with data from the 2010 perinatal study^37^. According to French, British, and Canadian recommendations, FGR was defined as a defect in fetal growth on two antenatal measurements taken two to three weeks apart^38–40^. FGR was retained according to the ICD10 codes in medical records (O36.5, P05.0, P05.1).

### Covariables

All variables available in the medical records were analyzed to detect potential confounders: maternal socioeconomic characteristics, obstetrical history, pregnancy complications and newborn characteristics (Supplementary Table S1).

Maternal age was calculated at delivery and dichotomized with a threshold of 35 years old. Maternal smoking during pregnancy was coded as “present” if active smoking was ticked in the medical records. Malnutrition was defined by pre-pregnancy body mass index lower than 18.5 or by the presence of an ICD10 code of malnutrition in medical records (O25, E43, E44). Obesity was defined by pre-pregnancy body mass index higher than 30. Vaginal bleeding in the second and third trimesters referred to an episode of bleeding after 28 weeks of gestation, including placental abruption and bleeding because of placenta previa (ICD10 codes: O45, O46, O441 and P021). Major infant congenital abnormalities corresponded to any major congenital anomalies according to the European network of population-based registries for the epidemiologic surveillance of congenital anomalies^41^. Infant congenital abnormalities considered for this study were determined before birth or at birth.

The neighborhood socioeconomic level was estimated with a collective socioeconomic index calculated at the geographical scale of the French sub-municipal census block groups (IRIS) defined by the National Institute of Statistic and Economics Studies^42^. Fifty IRIS in Besançon and 113 in the urban area of Dijon were included; population size ranged between 62 and 4811 inhabitants (mean = 2182 inhabitants). Variables related to family and household, immigration and mobility, employment and income, education and housing were extracted from the 2008 population census database. From among these variables, 39 were selected because of their occurrence in the literature^43–45^. The first component of a principal component analysis (PCA) was used to calculate a standardized socio-economic index following a reduction step. The socioeconomic index was calculated using the R package *SesIndexCreatoR*^43^. A value of the socioeconomic index in the last decile was considered as low neighborhood socioeconomic level.

### Environmental residential exposures

The residential exposure assessments have been previously described^10,33–35,46–50^. The participants’ addresses at the date of delivery were extracted from CPage software (version 2, https://www.cpage.fr/) using the personal identification number and the date of delivery. This address identified the residential building.

Two pollutants related to road traffic were studied. NO_2_ is a gaseous pollutant known to be the main indicator of road traffic^51^. Particulate matters (PM_10_) are also generated by road traffic and residential heating and were chosen because of their significant impact on human health and climate^52,53^. NO_2_ and PM_10_ exposure assessments were calculated at each mother’s building considering a 50 m radius buffer centered on the building centroid. The NO_2_ and PM_10_ levels were calculated using a two-step emission and diffusion modeling. Air pollutant emissions were calculated from road traffic data using CIRCUL'AIR software (version 2.51, www.atmo-grandest.eu), developed and used by all approved French Air Quality Monitoring Agencies (AASQA) (COPERT IV European standard methodology). AASQA’s pollution emission inventory was used to assess air pollutant emissions related to all activity sectors, especially heating, industries or agriculture. Air pollutant concentration was estimated 2 m above ground on a 25 m grid with reinforced gridding around the axes of emission, using the ADMS-Urban software (version 3.1.6, http://www.cerc.co.uk/) for diffusion modeling. ESRI ArcGIS software (version 10.1, https://www.esrifrance.fr/) was used for spatial interpolation to increase the spatial resolution of the ADMS output. Air pollutant concentration expressed in micrograms per cubic meter (μg/m^3^) was thus calculated at a 4m^2^ (2 m × 2 m) raster. The validity of the 2 m result was estimated on the basis of data from four, two-week-long measurement campaigns carried out during autumn and winter 2010 as well as spring and summer 2011. Measurements were based on 863 passive samplers and the nine AASQA air pollution measurement stations for NO_2_ (ATMO Franche-Comté and Atmosf’Air Bourgogne). Validation statistics (r^2^) range from 0.64 to 0.69. The validity of the PM_10_ models were verified using the city's fixed air-quality monitoring network. Monthly maps of air pollutant concentration were established from January 2004 to December 2009 using hourly meteorological data and background pollution levels to account for the seasonal variations in air pollutant concentrations. Using the monthly maps, time-weighted average air pollutant exposure was assessed over the following defined pregnancy periods: first, second and third trimester, entire pregnancy and two months before delivery.

Noise levels were calculated in accordance with the European Commission’s Environmental Noise Directive 2002/49/CE using an environmental noise prediction model with MithraSIG software (version 3.7, https://www.geomod.fr/fr/geomatique-modelisation-3d/mithrasig/)^54^. The following four types of noise sources were considered: road traffic, rail traffic, pedestrian streets, and fountains. Individual aircraft noise data were not available for the 2005–2009 period (military airport located within the city limits of Dijon). Women living in the area exposed to aircraft noise (according to the urban unit noise exposure plan) were not considered in this study. Theoretical noise levels were calculated in front of each building façade on each floor. Measurement campaigns were used for validation (76 points). The validation was good with a Spearman correlation coefficient at 0.81 (*p* < 0.01). For each woman, the average building noise levels in front of the entire façade were calculated using the following five indices: the daily equivalent A-weighted total noise level, L_Aeq,24 h_ for principal analysis; day equivalent A-weighted total noise level L_Aeq,day_ (6:00–18:00 h); evening equivalent A-weighted noise level L_Aeq,evening_ (18:00–22:00 h); night equivalent A-weighted total noise level L_Aeq,night_ (22:00–6:00 h); and combined day-evening-night A-weighted total noise level L_den_, with evening and night exposures penalized by 5 and 10 dB, respectively, for sensitivity analyses. Moreover, source-specific indices for L_Aeq,24 h_ were calculated for road and rail traffic related noise for sensitivity analyses.

### Statistics

The association between environmental exposures (NO_2_, PM_10_ and L_Aeq,24 h_) and SGA or FGR was estimated with simple and multiple logistic regression analyses, in single, and two-exposure models (air pollution with noise), where SGA or FGR were taken as binary outcomes in the models. Departure from the assumption of linearity was tested by introducing a polynomial function of the environmental exposure variables into the models. The ORs were adjusted for: term in gestational weeks, maternal age older than 35 years at delivery, low neighborhood socioeconomic level, maternal smoking during pregnancy, malnutrition and obesity, nulliparity, gestational hypertension, diabetes, assisted reproductive techniques, vaginal bleeding in the second and third trimesters, infection, and major infant congenital abnormalities. Because of the non-random distribution of missing data, a missing data class was used for categorical variables in multivariate analysis. Only two adjustment factors of the model had missing data: malnutrition and obesity (n = 219) and maternal smoking during pregnancy (n = 196), affecting only 2.4% of pregnancies. Potential interactions between air pollution and noise indices were assessed in models where at least one exposure was significant (*p*-value < 0.15). The interaction was tested using the *p*-value associated with the term from the regression analysis and comparing the Akaike Information Criterion (AIC) of models with and without the interaction term. Sensitivity analyses were conducted using four noise indices (L_Aeq,day_, L_Aeq,evening_, L_Aeq,night_, L_den_), two source-specific noise indices (road and rail traffic related noise L_Aeq,24 h_), and the other birth weight standard to define SGA as an outcome variable^37^. Maternal age at delivery and neighborhood socioeconomic level were also considered for adjustment in continuous form, or with a 2nd or 3rd order polynomial. Adjustment for year-season of conception was also considered in sensitivity analysis. Finally, a last analysis was conducted restricted to the subgroup of live births. Statistical analyses were performed with SAS software (version 9.4, SAS Institute, Cary, NC: https://www.sas.com/).

### Ethics

This study was approved by the French National Advisory Committee for the Treatment of Information in Health Research (CCTIRS) (registration number 15.292, April 9th 2015) and by the French data protection authority (CNIL) (registration number DR-2015-736, December 24th 2015). All methods were carried out in accordance with the ethical standards of CNIL and the Declaration of Helsinki. The requirement for patient informed consent was waived by the CNIL because of the retrospective nature of the study. A letter of information was sent to each eligible participant, and 20 families refused to participate. They were excluded from the study. All records were anonymized prior to analysis.

## Consent to participate

The requirement for patient informed consent was waived by the CNIL because of the retrospective nature of the study. So, in accordance with the CNIL recommendations, a letter of information was sent to each eligible family and those who refused to participate where not included.

## Results

Among the 10 570 single deliveries which occurred in the Besançon or Dijon university hospital from women over 18 years old and living in the defined study area, 8994 pregnancies were included in the study. Twenty pregnancies were excluded because the family opposed the use of their medical data, 103 due to incorrect address (wrong or unrecognizable recorded street names or anonymous childbirth) and 1453 pregnancies were excluded because the same woman had several pregnancies over the study period.

Among the 8994 pregnancies, 587 had FGR and 918 had SGA. Five hundred and five (55.0%) fetuses with SGA did not have FGR; and 172 (29.4%) fetuses with FGR did not have SGA. SGA and FGR were significantly associated (*p*-value < 0.0001, Chi-square test). The pregnancy and newborn characteristics are presented in Table 1.

**Table 1:** Pregnancy and newborn characteristics according to fetal growth restriction and small for gestational age status, 2005–2009 (N = 8994).

	N	Total	Small for gestational age^1^		Fetal growth restriction	
		*N (%)*	Yes *N (%)*	No *N (%)*	Yes *N (%)*	No *N (%)*
**Pregnancies**		N = 8994	N = 918	N = 8071	N = 587	N = 8407
City of residence	8994					
Besançon		3684 (41.0)	433 (47.2)	3246 (40.2)	257 (43.8)	3427 (40.8)
Dijon		5310 (59.0)	485 (52.8)	4825 (59.8)	330 (56.2)	4980 (59.2)
Maternal age at delivery > 35 years old	8994	1424 (15.8)	130 (14.2)	1292 (16.0)	85 (14.5)	1339 (15.9)
Low neighborhood socioeconomic level	8994	1308 (14.5)	142 (15.5)	1165 (14.4)	82 (14.0)	1226 (14.6)
Living status	8577					
Living alone		716 (8.4)	104 (11.8)	612 (8.0)	66 (11.8)	650 (8.1)
Married, cohabitation, others		7861 (91.6)	779 (88.2)	7077 (92.0)	495 (88.2)	7366 (91.9)
Maternal employment during pregnancy	8504	5455 (64.2)	540 (62.1)	4913 (64.4)	352 (63.0)	5103 (64.2)
Maternal smoking during pregnancy	8798	1665 (18.9)	318 (35.0)	1346 (17.1)	207 (36.0)	1458 (17.7)
Pre-pregnancy body mass index (kg/m^2^)	8775					
< 25		6226 (70.9)	694 (77.5)	5527 (70.2)	457 (79.9)	5769 (70.3)
25–30		1728 (19.7)	133 (14.8)	1595 (20.3)	80 (14.0)	1648 (20.1)
30 (obesity)		821 (9.4)	69 (7.7)	752 (9.5)	35 (6.1)	786 (9.6)
Malnutrition	8775	671 (7.7)	122 (13.6)	549 (7.0)	82 (14.3)	589 (7.2)
Nulliparity	8994	4809 (53.5)	593 (64.6)	4212 (52.2)	392 (66.8)	4417 (52.5)
History of medical interruption of pregnancy	8922	130 (1.5)	13 (1.4)	117 (1.5)	9 (1.5)	121 (1.5)
History of preterm delivery	8786	164 (1.9)	18 (2.0)	146 (1.9)	12 (2.1)	152 (1.9)
Abnormalities of the female reproductive tract	8994	722 (8.0)	62 (6.8)	658 (8.2)	49 (8.4)	673 (8.0)
Uterine scar	8994	617 (6.9)	50 (5.5)	566 (7.0)	41 (7.0)	576 (6.9)
Assisted reproductive techniques	8994	188 (2.1)	21 (2.3)	167 (2.1)	19 (3.2)	169 (2.0)
Gestational hypertension	8994	382 (4.3)	74 (8.1)	308 (3.8)	79 (13.5)	303 (3.6)
Vaginal bleeding in the second and third trimesters	8994	222 (2.5)	32 (3.5)	190 (2.4)	29 (4.9)	193 (2.3)
Placental abruption	8994	71 (0.8)	15 (1.6)	56 (0.7)	15 (2.6)	56 (0.7)
Placenta previa	8994	57 (0.6)	6 (0.7)	51 (0.6)	4 (0.7)	53 (0.6)
Hemorrhagic placenta previa	8994	33 (0.4)	3 (0.3)	30 (0.4)	1 (0.2)	32 (0.4)
Infection	8994	1258 (14.0)	135 (14.7)	1123 (13.9)	93 (15.8)	1165 (13.9)
Infection of amniotic fluid	8994	84 (0.9)	8 (0.9)	76 (0.9)	10 (1.7)	74 (0.9)
Genitourinary infection	8994	979 (10.9)	99 (10.8)	880 (10.9)	58 (9.9)	921 (11.0)
Diabetes	8994	691 (7.7)	50 (5.5)	641 (7.9)	34 (5.8)	657 (7.8)
Hydramnios	8994	116 (1.3)	6 (0.7)	110 (1.4)	5 (0.9)	111 (1.3)
Premature rupture of membranes	8994	1173 (13.0)	91 (9.9)	1081 (13.4)	76 (13.0)	1097 (13.1)
Prematurity (≤ 36 SA)	8994	736 (8.2)	92 (10.0)	643 (8.0)	128 (21.8)	608 (7.2)
**Newborns**						
Status	8994					
Living		8883 (98.8)	886 (96.5)	7993 (99.0)	577 (98.3)	8306 (98.8)
Stillborn		98 (1.1)	30 (3.3)	67 (0.8)	9 (1.5)	89 (1.1)
Deceased shortly after birth		13 (0.1)	2 (0.2)	11 (0.2)	1 (0.2)	12 (0.1)
Sex	8991					
Male		4686 (52.1)	470 (51.2)	4214 (52.2)	251 (42.9)	4435 (52.8)
Female		4305 (47.9)	448 (48.8)	3857 (47.8)	334 (57.1)	3971 (47.2)
Birth weight (g)	8992	3215 (597)	2494 (515)	3298 (548)	2360 (546)	3275 (553)
Major infant congenital abnormalities	8994	305 (3.4)	32 (3.5)	270 (3.4)	35 (6.0)	270 (3.2)
Apgar score at five minutes = 10	8833	8008 (90.7)	787 (88.7)	7218 (90.9)	485 (86.0)	7523 (91.0)

N: number; N (%): number (percentage) except for birth weight which is described by mean (standard deviation).

^1^Lower than 10th centile of birth weight for gestational age.

The median NO_2_ concentration considering a 50 m radius buffer during the entire pregnancy was 23.2 µg/m^3^; the minimum exposure was 7.4 µg/m^3^ and the maximum exposure was 51.6 µg/m^3^ (Supplementary Figure S1, Supplementary Table S2). The median PM_10_ concentration considering a 50 m radius buffer during the entire pregnancy was 18.5 µg/m^3^; the minimum exposure was 11.9 µg/m^3^ and the maximum exposure was 31.5 µg/m^3^ (Supplementary Figure S2, Supplementary Table S2). The median daily equivalent A-weighted total noise level was 55.5 dB; the minimum exposure was 33.2 dB and the maximum exposure was 76.9 dB (Supplementary Figure S3, Supplementary Table S2). The Pearson correlation coefficients for environmental exposure were: 0.56 between L_Aeq,24 h_ and NO_2_ exposure during the entire pregnancy, 0.28 between L_Aeq,24 h_ and PM_10_ exposure during the entire pregnancy, and 0.28 between NO_2_ and PM_10_ exposure during the entire pregnancy (*p*-value < 10^–3^) (Supplementary Table S3).

We found no departure from the assumption of linearity of pregnancy outcomes with noise and air pollution (Supplementary Figure S4).

For SGA, the crude OR associated with a 5 dB increase of total building L_Aeq,24 h_ was 0.97 (95% CI 0.91–1.03) (Table 2). The adjusted OR remained similar in single and two-exposure models. The crude OR associated with a 10 µg/m^3^ increase in NO_2_ exposure during the first, second and third trimester, the entire pregnancy and the two months before delivery were 0.94 (95% CI 0.86–1.02), 0.96 (95% CI 0.87–1.04), 0.95 (95% CI 0.87–1.04), 0.94 (95% CI 0.86–1.03), and 0.96 (95% CI 0.88–1.05), respectively. The adjusted OR remained similar in single-exposure models and in two-exposure models with total building L_Aeq,24 h_. The crude OR associated with a 10 µg/m^3^ increase in PM_10_ exposure during the first, second and third trimester, the entire pregnancy and the two months before delivery were 1.02 (95% CI 0.86–1.22), 1.17 (95% CI 0.99–1.39), 1.21 (95% CI 1.03–1.43), 1.29 (95% CI 1.00–1.66), and 1.20 (95% CI 1.02–1.41), respectively. The adjusted OR in single-exposure models decreased slightly and became non-significant. In the two-exposure models with total building L_Aeq,24 h_, only the adjusted OR associated with a 10 µg/m^3^ increase in PM_10_ exposure during the two months before delivery was significant: 1.18 (95% CI 1.00–1.40). Sensitivity analyses were conducted using the birth weight standard of Ego (Cf. Material and methods). The proportion of pregnancies with SGA was 12.4%. Sensitivity analyses led to OR estimates close to the main analysis.

**Table 2:** Relationship between noise, NO_2_ and PM_10_ exposure during pregnancy and small for gestational age or fetal growth restriction, 2005–2009 (N = 8994).

	Outcome		OR [95% CI] for an increase of 5 dB or 10 µg/m^3^					
	Yes *µ (SD)*	No *µ (SD)*	Single-exposure models				Two-exposure noise and air pollution models	
			Crude OR	*p*-value^1^	Adjusted OR^2^	*p*-value^1^	Adjusted OR^2^	*p*-value^1^
**Small for gestational age**	N = 918	N = 8071	N = 8989		N = 8989		N = 8989	
Total noise exposure (dB) building L_Aeq,24 h_							1.00 [0.92; 1.08]^6^	0.89
Entire pregnancy^3^	55.4 (5.5)	55.6 (5.4)	0.97 [0.91; 1.03]	0.30	0.97 [0.91; 1.03]	0.31	0.95 [0.89; 1.02]^7^	0.15
NO_2_ concentration, 50 m radius buffer (µg/m^3^)								
First trimester	24.3 (8.0)	24.7 (7.8)	0.94 [0.86; 1.02]	0.15	0.92 [0.84; 1.01]	0.09	0.93 [0.83; 1.03]	0.16
Second trimester	24.2 (8.0)	24.5 (7.7)	0.96 [0.87; 1.04]	0.31	0.93 [0.85; 1.02]	0.14	0.94 [0.85; 1.05]	0.27
Third trimester^4^	24.2 (7.9)	24.5 (7.7)	0.95 [0.87; 1.04]	0.25	0.93 [0.85; 1.02]	0.13	0.93 [0.84; 1.04]	0.22
Entire pregnancy	24.2 (7.6)	24.5 (7.4)	0.94 [0.86; 1.03]	0.20	0.92 [0.84; 1.02]	0.10	0.93 [0.83; 1.04]	0.18
The two months before delivery	24.2 (8.0)	24.5 (7.8)	0.96 [0.88; 1.05]	0.34	0.94 [0.86; 1.03]	0.19	0.95 [0.86; 1.06]	0.36
PM_10_ concentration, 50 m radius buffer (µg/m^3^)								
First trimester	18.7 (3.9)	18.7 (3.9)	1.02 [0.86; 1.22]	0.80	1.00 [0.84; 1.20]	0.97	1.02 [0.85; 1.23]	0.81
Second trimester	18.8 (3.9)	18.6 (3.9)	1.17 [0.99; 1.39]	0.07	1.11 [0.93; 1.32]	0.26	1.13 [0.95; 1.36]	0.18
Third trimester^4^	18.9 (4.0)	18.6 (4.0)	1.21 [1.03; 1.43]	0.02	1.16 [0.98; 1.38]	0.09	1.19 [1.00; 1.41]	0.06
Entire pregnancy	18.8 (2.6)	18.6 (2.7)	1.29 [1.00; 1.66]	0.05	1.18 [0.91; 1.53]	0.21	1.25 [0.95; 1.64]	0.11
The two months before delivery	18.9 (4.1)	18.5 (4.2)	1.20 [1.02; 1.41]	0.03	1.16 [0.99; 1.37]	0.08	1.18 [1.00; 1.40]	0.05
**Fetal growth restriction**	N = 587	N = 8407	N = 8994		N = 8994		N = 8994	
Total noise exposure (dB) building L_Aeq,24 h_							0.99 [0.90; 1.09]^6^	0.80
Entire pregnancy^3^	55.5 (5.5)	55.6 (5.4)	0.98 [0.90; 1.06]	0.55	0.98 [0.90; 1.06]	0.58	0.98 [0.90; 1.06]^7^	0.61
NO_2_ concentration, 50 m radius buffer (µg/m^3^)								
First trimester	24.2 (7.7)	24.7 (7.8)	0.93 [0.83; 1.03]	0.18	0.92 [0.82; 1.03]	0.13	0.91 [0.80; 1.04]	0.15
Second trimester	24.4 (7.8)	24.4 (7.7)	1.00 [0.90; 1.11]	0.97	0.98 [0.87; 1.10]	0.71	0.99 [0.87; 1.13]	0.91
Third trimester^5^	24.6 (7.7)	24.4 (7.7)	1.03 [0.93; 1.15]	0.56	1.01 [0.90; 1.13]	0.93	1.04 [0.91; 1.19]	0.54
Entire pregnancy	24.4 (7.4)	24.5 (7.4)	0.98 [0.88; 1.10]	0.73	0.96 [0.86; 1.08]	0.54	0.97 [0.85; 1.12]	0.70
The two months before delivery	24.7 (7.8)	24.4 (7.8)	1.04 [0.93; 1.16]	0.49	1.03 [0.92; 1.15]	0.66	1.06 [0.93; 1.20]	0.41
PM_10_ concentration, 50 m radius buffer (µg/m^3^)								
First trimester	18.4 (3.8)	18.7 (3.9)	0.78 [0.63; 0.98]	0.03	0.77 [0.61; 0.97]	0.02	0.77 [0.61; 0.97]	0.03
Second trimester	18.6 (3.8)	18.6 (3.9)	1.02 [0.82; 1.26]	0.87	0.96 [0.77; 1.20]	0.71	0.97 [0.77; 1.22]	0.78
Third trimester^5^	19.1 (4.3)	18.5 (4.0)	1.39 [1.14; 1.70]	< 0.01	1.35 [1.09; 1.66]	< 0.01	1.38 [1.12; 1.70]	< 0.01
Entire pregnancy	18.7 (2.7)	18.6 (2.7)	1.05 [0.77; 1.43]	0.77	0.97 [0.70; 1.33]	0.85	0.99 [0.71; 1.39]	0.97
The two months before delivery	19.1 (4.4)	18.5 (4.2)	1.35 [1.11; 1.63]	< 0.01	1.33 [1.09; 1.62]	< 0.01	1.35 [1.11; 1.66]	< 0.01

N: number; µ (SD): exposure average (standard deviation); OR: Odds ratio; CI: confidence interval.

^1^Wald Chi-square test.

^2^Adjusted for term, maternal age above 35 years at delivery, low neighborhood socioeconomic level, maternal smoking during pregnancy, malnutrition and obesity, nulliparity, gestational hypertension, diabetes, assisted reproductive techniques, vaginal bleeding in the second and third trimesters, infection, major infant congenital abnormalities.

^3^For NO_2_ and PM_10_ indices in adjusted analyses (the results were similar with the other period indices).

^4^Missing data for delivery before 29 weeks of gestational age (n = 106).

^5^Missing data for delivery before 29 weeks of gestational age (n = 107).

^6^Noise and NO_2_.

^7^Noise and PM_10_.

When considering FGR, the crude OR associated with a 5 dB increase of total building L_Aeq,24 h_ was 0.98 (95% CI 0.90–1.06) (Table 2). The adjusted OR remained similar in single and two-exposure models. The crude OR associated with a 10 µg/m^3^ increase in NO_2_ exposure during the first, second and third trimester, the entire pregnancy and the two months before delivery were 0.93 (95% CI 0.83–1.03), 1.00 (95% CI 0.90–1.11), 1.03 (95% CI 0.93–1.15), 0.98 (95% CI 0.88–1.10), and 1.04 (95% CI 0.93–1.16), respectively. The adjusted OR remained similar in single and two-exposure models with values close to 1. The crude OR associated with a 10 µg/m^3^ increase in PM_10_ exposure during the first, second and third trimester, the entire pregnancy and the two months before delivery were 0.78 (95% CI 0.63–0.98), 1.02 (95% CI 0.82–1.26), 1.39 (95% CI 1.14–1.70), 1.05 (95% CI 0.77–1.43), and 1.35 (95% CI 1.11–1.63), respectively. The adjusted OR remained similar in single and two-exposure models.

For SGA, the OR for single-exposure adjusted models for an increase of an IQR were 0.95 (95% CI 0.87–1.05) for total building L_Aeq,24 h_, 0.92 (95% CI 0.84–1.02) for NO_2_ exposure during the third trimester, and 1.08 (95% CI 0.99–1.18) for PM_10_ exposure during the third trimester, respectively. For FGR, they were 0.97 (95% CI 0.87–1.09) for building L_Aeq,24 h_, 1.01 (95% CI 0.89–1.14) for NO_2_ exposure during the third trimester, and 1.16 (95% CI 1.05–1.29) for PM_10_ exposure during the third trimester, respectively.

The two centers differed on characteristics of the pregnancy, for example: low neighborhood socioeconomic level (16.2 for Besançon versus 13.4% for Dijon), malnutrition (9.1% for Besançon versus 6.6% for Dijon), prematurity (7.3% for Besançon versus 8.8% for Dijon), or small for gestational age (11.8% for Besançon versus 9.1% for Dijon), but not on FGR (7.0% for Besançon versus 6.2% for Dijon). Environmental exposures were significantly different between the two centers: in Besançon, noise and NO_2_ levels were lower, but PM_10_ levels were higher (Supplementary Table S4). Adjustment for the maternity of delivery has been tested and stratified analyses on the maternity of delivery have been performed. In the adjusted analysis, OR associated with total noise exposure remained similar and did not change the OR associated with air pollution exposure in the two-exposure models. The results were also similar in the stratified analyses. For example, for FGR, OR associated with a 5 dB increase of total building L_Aeq,24 h_ was 0.93 (95% CI 0.82–1.05) for Besançon and 1.03 (95% CI 0.92–1.15) for Dijon, and OR associated with a 10 µg/m^3^ increase in PM_10_ exposure during the third trimester was 1.33 (95% CI 1.00–1.75) for Besançon and 1.30 (95% CI 0.93–1.81) for Dijon in single exposure models. And OR associated with a 10 µg/m^3^ increase in PM_10_ exposure during the third trimester were 1.41 (95% CI 1.06–1.88) for Besançon and 1.31 (95% CI 0.93–1.85) for Dijon in two exposure models.

Interactions between noise and air pollution do not improve the adequacy of the models. Furthermore, no synergistic or antagonist effects were observed in the models with the interaction term.

Sensitivity analyses led to similar results when considering road or rail traffic related noise exposure, maternal age or neighborhood socioeconomic level in continuous variable with 1st, or a 2nd or a 3rd order polynomial, or restricted to the subgroup of live births (Supplementary Tables S5, S6, S7, S8, and S9). In the sensitivity analysis with adjustment for year-season of conception, the OR for the association between first trimester PM_10_ concentration and FGR became non-significant and the other estimates did not been change (Supplementary Table S10).

## Discussion

Beyond studies with high levels of noise and air pollution, the situation with low levels of air pollution makes it possible to better assess the effect of noise on the relationship between air pollution and fetal growth disorders, avoiding a potential masking effect by high levels of air pollution. The systematic two-exposure modeling allowed to explore for confounding of noise on the relationship between air pollution and fetal growth. In our study, we first confirmed the previously observed association between moderate exposure to PM_10_ and fetal growth disorders, especially towards the end of pregnancy. Our results are also in favor of a lack of association between environmental noise exposure and fetal growth disorders in singletons. Finally, our data suggest that moderate exposure to noise has no confounding or modifying effect on the relationship between fetal growth and environmental exposure to the air pollution analyzed.

### Air pollution, noise and fetal growth

In a context of moderate levels of air pollution, our results are in favor of an independent association between air pollution, and of a lack of association between noise and NO_2_ exposure and fetal growth disorders.

#### Air pollution and fetal growth

Most of the studies that found that air pollution (NO_2_ or PM_10_) had a negative impact on fetal growth (SGA or FGR) were conducted on more polluted areas than in our study (1.5 to twofold more for NO_2_ and 2 to threefold more for PM_10_)^21,55–60^. Our results confirm that exposure to PM_10_ at the end of the pregnancy has a negative impact on FGR in a moderately polluted area. However, it could not be excluded that the study of finer particles would have led to different results (data were unfortunately not available for our study area). A study conducted in Vancouver, Canada, where the levels of NO_2_ were comparable to those of our study area, also described a negative impact of NO_2_ on SGA^61^. Other studies found no associations between NO_2_ and SGA^27,62–65^. The OR in our study ranged between 0.92 and 0.96 and were not significant. We conducted a precedent study in a population of multiple pregnancies (twins and triplets) and found no association between NO_2_ exposure and SGA with non-significant OR ranged between 0.78 and 0.88, but an association between NO_2_ exposure and FGR with OR ranged between 1.42 and 1.52^35^.

#### Noise and fetal growth

In agreement with our results, a recent meta-analysis conducted by Dzhambov and Lercher found no association between noise and SGA (OR for an increase of 10 dB(A) = 1.02; 95% CI 0.86, 1.21) (I^2^ = 90%)^66^. This meta-analysis was performed on five populations in four cohort or cross-sectional studies^27,29,32,67^. However, levels of noise exposure were moderate in our study and associations between noise and fetal growth disorders could be plausible at higher levels of exposure.

#### Multiexposure to noise and air pollution, and fetal growth

Our study is one of the rare studies exploring co-exposure to environmental noise and air pollution. Other studies have not specifically explored the influence of noise on the relationship between air pollution and fetal growth disorders, and have used different methods in contexts different from ours. In the metropolitan area of Vancouver, Canada, Gehring et al. found an association between all transportation noise exposure and SGA. The observed association between NO_2_ exposure and SGA differed according to the assessment of exposure: the OR associated with an IQR increase was 0.98 (95% CI 0.96–1.01) when a land-use regression model was used and 1.10 (95% CI 1.06–1.15) for the inversed distance weighting method. In co-pollutant models, only noise was associated with SGA^29^. In two surveys in the Alpine area, a positive association between noise and SGA was revealed in two-exposure model with adjustment for NO_2_ exposure, which was not associated with SGA in the first survey. In the second survey, noise was negatively associated with SGA though this association disappeared with adjustment for NO_2_ exposure^67^. In London, Smith et al. found no association between NO_2_, PM_10_, noise and SGA in one and co-pollutant models^32^.

Other studies analyze the association between noise, air pollution and (term-) birth weight or (term-) LBW or birth weight^26–28,30,31^. Two ecological time-series studies in Madrid, Spain, assessed the impact of PM_2.5_, NO_2,_ O_3_, noise, and temperature on LBW and found an impact on LBW of: (1) daily diurnal noise at onset of gestation, in the second trimester and in the week of birth itself, and of NO_2_ in the second trimester, in all live singleton births for the first study; (2) only of PM_2.5_ in non-preterm births for the second study^26,28^. A cohort study in Barcelona, Spain, based on singleton term births found no effect of noise but an effect of PM_2.5_ and PM_10_ in the third trimester on LBW^27^. In a study on the Danish National Birth Cohort, no association between road traffic noise and NO_2_ on birth weight was found in children born at term in two exposure models^30^. Finally, a study grouping six European birth cohorts found no association between NO_2_, PM_10_, PM_2.5_, road traffic noise and LBW^31^.

### Potential biases, study limits and strengths

The negative association between PM_10_ exposure during the first trimester and FGR could be explained by the combined effects of three factors: the effect of the exposure on fetal growth, the seasonal variations of the exposure, and the existence of a gestational period of sensitivity to exposure. In fact, PM_10_ levels vary in a 1-year cycle and are higher in the colder season than in warmer season due to anthropic activities (transport and heating). The seasonal variations are higher for PM_10_ than NO_2_. The duration of a pregnancy is 9 months. So when the first trimester of a pregnancy is in winter, then the third trimester will be in summer; PM_10_ levels will be higher in the first trimester than in the third. Conversely, when the first trimester of a pregnancy is in summer, the third will be in winter; PM_10_ levels will be higher in the third trimester than in the first. This is consistent with the sensitivity analysis adjusted for year-season of conception where the OR for the first trimester became non-significant. The mechanism of placental hypoperfusion intervenes especially at the end of pregnancy on fetal growth. In the hypothesis of an impact of PM_10_ on fetal growth, women with a third trimester in winter may experience more frequently FGR than women with a third trimester in summer. Higher PM_10_ levels will be associated with the occurrence of FGR, the observed OR may be greater than one for the third trimester exposure. Conversely, for women with a first trimester in the summer associated with lower PM_10_ levels, the observed OR may be lower than one for the first trimester exposure. This could potentially lead to seasonality bias. Moreover, as suggested by Hao et al. and by Raz et al., we cannot exclude a selection bias called live-birth bias at the beginning of the pregnancy due to early abortions that can be caused by exposure to air pollution because they were not detectable in our study and therefore were not included^68,69^. Raz et al. described two types of live-birth bias: (1) the selection of a group among whom those with high exposure to air pollution could have low exposure to other risk factors for FGR (or higher exposure to protective factors), or (2) the selection of a group among whom those with high exposure to air pollution could have lower susceptibility to the effects of other risk factors for FGR than those with lower air-pollution exposure.

Compared to the single pregnancies recorded in the 2010 French perinatal study, our study sample presented just slightly adverse outcomes^70^. In fact, the two maternities included are level 3 maternity units where complicated pregnancies are more closely monitored. But the two public university hospitals included in the study are also obstetrical primary care hospitals. So because of their immediate proximity for women living in the studied urban areas, the effect of the reference status of the two maternities is limited.

SGA and FGR seem to be the most relevant indicators of fetal growth abnormalities. In fact, the sexually dimorphic differences in growth of the fetus is mediated by the sex specific function of the human placenta^71^ and SGA and FGR take into account the sex of the fetus. They were defined differently. On one hand, SGA is defined as a weight lower than the 10th centile of weight for gestational age and sex. This indicator was objectively determined from the French perinatal network reference of birth weight for sex and gestational age^36^. When it was compared with another reference; the associations were similar. On the other hand, FGR was established from the ICD10 codes listed in medical records. FGR is defined as a defect in fetal growth on two antenatal measurements two to three weeks apart according to French, British, and Canadian recommendations^38–40^. FGR is rarely used as an outcome in environmental epidemiological studies because it is more difficult to diagnose FGR retrospectively than SGA^58^. However, SGA is defined by birth weight at gestational age and sex and the size and weight of newborns are strongly influenced by those of their parents. Moreover, SGA does not take into account the growth trajectory as opposed to FGR, which is a dynamic measure of growth regardless of the measured weight value. So SGA could be less specifically a disorder of fetal growth than FGR, and seems less appropriate for identifying an association between fetal growth disorders and environmental exposure than FGR. A coding effect cannot be ruled out because FGR implies a dynamic evaluation of fetal growth during pregnancy by obstetricians. However, this potential coding effect was reduced by the multicentric quality of this study.

Specific attention was paid to the collection of data from medical records. The consultation of paper medical records resulted in the collection of potential confounding factors for the adjustment of the analyses. Data was missing for two adjustment factors of the model (malnutrition/obesity and maternal smoking during pregnancy) but involved only 2.4% of pregnancies.

Another limit was the absence of information about a potential move during pregnancy – we used the mother's address at delivery, which was recorded in the hospital information system upon admission, for geocoding.

Individuals spend about 80% of the time in indoor environments (European Commission, 2004) and French women spend 16/24 h (67% of the time) inside their dwelling^72^, but we did not use indoor air measurements for our study. Due to the retrospective design of this study, NO_2_ exposure was assessed using modelled outdoor exposure. However, a study in Vancouver, Canada, found a good agreement between indoor air measurements and outdoor values obtained by modeling^73,74^; a similar approach previously conducted by our team in the study area led to similar conclusions^46,47^; furthermore the retrospective modeling of exposure allowed for repeatable assessment of exposure. We assessed only residential exposure but time spent at home increases during maternity leave. We cannot however exclude exposure misclassification; but the definition of the air pollution and noise indicators that we used in this study has been explored in precedent works of our team^49,50^. Particular attention was paid to calculate NO_2_ and PM_10_ exposure closest to the home in the immediate neighborhood^50^. We used the average noise of all façades because the use of the noise of the most (least) exposed façade could induce an over (under) estimation of the exposure. Furthermore, the closer from the main source the building is located, the higher the overestimation could be. So when considering individual exposure, the most exposed façade could lead to differential error measurement. And last, this influence of the proximity of the source on the error assessment is more marked for noise than for air pollution. Moreover, we do not know the floor and the façade of the dwelling of the women included in our study. In one of the two areas, 25% of the buildings are very close to a main road. All these points could lead to a significant overestimation of the noise exposure of the most exposed buildings^49^, and then to a high risk of differential error assessment between dwelling, and between noise and air pollution exposure.

## Conclusions

The study confirms a positive association between fetal growth disorders in single pregnancies and environmental exposure to air pollution at the end of pregnancy, in moderately polluted cities. In these conditions of moderate noise exposure, no argument in favor of a noise effect on the relationship between fetal growth and environmental exposure to air pollution was found in our study. These results need to be confirmed in areas with high levels of noise so as not to overlook the effect of high exposure of noise on the relationship between air pollution and fetal growth disorders.

## Supplementary information

Supplementary Information.

## Data Availability

In accordance with the CNIL recommendations and the letter of information sent to each eligible family, we are not authorized to transmit the data.
