# Supplementary material for: Association between moderated level of air pollution and fetal growth: the potential role of noise exposure
Source: Sci Rep. 2021 May 27;11:11238. doi: 10.1038/s41598-021-90788-1 (PMC8160128; doi:10.1038/s41598-021-90788-1)

Supplementary Tables and figures

Article entitled "Association between moderated level of air pollution and fetal growth: the potential role of noise exposure"

Name of the authors
Anne-Sophie MARIET[1,2,3*], Nadine BERNARD[4,5], Sophie PUJOL[4,6], Paul SAGOT[7], Gérard THIRIEZ[8], Didier RIETHMULLER[9], Mathieu BOILLEAUT[10], Jérôme DEFRANCE[11], Hélène HOUOT[5], Anne-Laure PARMENTIER[4,6], Eric BENZENINE[1,2], Frédéric MAUNY[4,6], Catherine QUANTIN[1,2,3]

Affiliations and address of the authors
1 CHU Dijon Bourgogne, Service de Biostatistiques et d'Information Médicale, F-21000 Dijon, France
2 CHU Dijon Bourgogne, Inserm, Clinical Investigation Center of Dijon (Inserm CIC 1432), F-21000 Dijon, France
3 Université Bourgogne Franche-Comté, Inserm, Biostatistique, Biomathématique, Pharmacoépidémiologie et Maladies Infectieuses (B2PHI), UMR 1181, F-21000 Dijon, France
4 Université de Bourgogne Franche-Comté, CNRS, Laboratoire Chrono-environnement UMR 6249, F-25000 Besançon, France
5 Université de Bourgogne Franche-Comté, CNRS, Laboratoire ThéMA UMR 6049, F-25000 Besançon, France
6 CHU de Besançon, Unité de méthodologie en recherche clinique, épidémiologie et santé publique, INSERM CIC 1431, F-25000 Besançon, France
7 CHU Dijon Bourgogne, Service de Gynécologie-Obstétrique, F-21000 Dijon, France
8 CHU de Besançon, Service de Réanimation Pédiatrique, Néonatalogie et Urgences Pédiatriques, F-25000 Besançon, France
9 CHU de Besançon, Service de Gynécologie-Obstétrique, F-25000 Besançon, France
10 Atmo Bourgogne-Franche-Comté, F-25000 Besançon, France
11 Centre Scientifique et Technique du Bâtiment, Pôle Acoustique et Eclairage, F-38400 Saint Martin d'Hères, France

E-mail address and telephone number of the corresponding author
Dr. Anne-Sophie MARIET, e-mail: anne-sophie.mariet@chu-dijon.fr

****

List of supplementary tables and figures

Table S1. Precisions about definitions and/or associated ICD-10 codes of covariables

Table S2. Description of environmental exposures for all pregnancies and between groups of fetal growth disorders: small for gestational age or fetal growth restriction (N = 8994)

Table S3. Pearson's correlation coefficients between environmental exposures for all pregnancies (N = 8994)

Table S4. Environmental exposures according to the city of residence, 2005–2009 (N = 8994)

Table S5. Sensitivity analyses on noise exposure sources. Relationship between total noise, road traffic related noise, rail traffic related noise, NO_2 and PM_{10} exposure during pregnancy and small for gestational age or fetal growth restriction, 2005-2009 (N = 8994)

Table S6. Sensitivity analyses on adjustment for maternal age at delivery. Relationship between total noise, NO_2 and PM_{10} exposure during pregnancy and small for gestational age or fetal growth restriction, with maternal age at delivery considered for adjustment in continuous form, or with a second or third order polynomial, 2005-2009 (N = 8994)

Table S7. Sensitivity analyses on adjustment for neighborhood socioeconomic level. Relationship between total noise, NO_2 and PM_{10} exposure during pregnancy and small for gestational age or fetal growth restriction, with neighborhood socioeconomic level considered for adjustment in continuous form, or with a second or third order polynomial, 2005-2009 (N = 8994)

Table S8. Sensitivity analysis on live births only. Pregnancy and newborn characteristics according to fetal growth restriction and small for gestational age status, 2005–2009 (N = 8994)

Table S9. Sensitivity analysis on live births only. Relationship between total noise, NO_2 and PM_{10} exposure during pregnancy and small for gestational age or fetal growth restriction, 2005-2009 (N = 8994)

Table S10. Sensitivity analysis with adjustment for year-season of conception. Relationship between noise, NO_2 and PM_{10} exposure during pregnancy and small for gestational age or fetal growth restriction, 2005-2009 (N = 8994)

****

Figure S1. NO_2 exposure, considering a 50m radius buffer, during different periods of pregnancy, according to fetal growth restriction and small for gestational age status, 2005–2009 (N=8994)

Figure S2. PM_{10} exposure, considering a 50m radius buffer, during different periods of pregnancy, according to fetal growth restriction and small for gestational age status, 2005–2009 (N=8994)

Figure S3. Total noise exposure, considering the average building noise levels in front of the entire façade (daily, day, evening, night equivalent, and day-evening-night A-weighted noise level), according to fetal growth restriction and small for gestational age status, 2005–2009 (N=8994)

Figure S4. Representation of the Logit of the observed probability of fetal growth restriction or small for gestational age, according to total noise, NO_2 and PM_{10} exposure during the third trimester, 2005-2009 (N = 8994)

****

Table S1. Precisions about definitions and/or associated ICD-10 codes of covariables

Variables	Precisions about definitions and/or associated ICD-10 codes
**PREGNANCIES**	
City of residence	City of residence at delivery
- Besançon	
- Dijon	
Maternal age at delivery > 35 years old	In the medical records
Low neighborhood socioeconomic level	Value of the socioeconomic index in the last decile
Living status	Declared lifestyle in the medical records
- Living alone	
- Married, cohabitation, others	
Maternal employment during pregnancy	In the medical records
Maternal smoking during pregnancy	Active smoking ticked in the medical records
Pre-pregnancy body mass index (BMI) (kg/m²)	BMI before pregnancy in the medical records
- < 25	
- 25 – 30	
- > 30 (obesity)	
Malnutrition	Pre-pregnancy BMI < 18.5 or O25, E43, E44
Nulliparity	Parity at 0 before the included pregnancy
History of medical interruption of pregnancy	In the medical records
History of preterm delivery	In the medical records
Abnormalities of the female reproductive tract	O34, [Q50-Q52]
Uterine scar	O34.2
Assisted reproductive techniques	In the medical records
Gestational hypertension	[I10-I15], [O10-O11], [O13-O16], P00.0
Vaginal bleeding in the second and third trimesters	[O45-O46], O44.1, P02.1
Placental abruption	O45
Placenta previa	O44, P02.0
Hemorrhagic placenta previa	O44.1
Infection	O23, O98, Z21, A34, [A15-A19], [A50-A64], [B15-B19], [A80-B09], [B25-B34], Z22.5, P00.2
Infection of amniotic fluid	O41.1
Genitourinary infection	O23, A181, A51.0, [A54.0-A54.2], [A56.0-A56.2], A59.0, A60.0
Diabetes	[E10-E14], O24, [P70.0-P70.1]
Hydramnios	O40
Premature rupture of membranes	O42, O75.6
Prematurity (≤ 36 SA)	Term ≤ 36 weeks of gestation
**NEWBORNS**	
Status	In the medical records
- Living	
- Stillborn	
- Deceased shortly after birth	
Sex	In the medical records
- Male	

- Female	
Birth weight (g)	In the medical records
Fetal growth restriction	O36.5, P05.0, P05.1
Small for gestational age	Birth weight < 10th centile for gestational age and sex
Major infant congenital abnormalities	Any major congenital anomalies according to the European network of population-based registries for the epidemiologic surveillance of congenital anomalies (EUROCAT, 2005)
Apgar score at five minutes = 10	In the medical records

[XXX-YYY]: codes XXX to YYY were included.

Table S2. Description of environmental exposures for all pregnancies and between groups of fetal growth disorders: small for gestational age or fetal growth restriction (N = 8994)

Variable	Group	N	Mean	SD	Minimum	Q1	Median	Q3	Maximum
Total noise exposure (dB) building									
- $L_{Aeq,24h}$	All	8994	55.6	5.4	33.2	52.2	55.5	59.2	76.9
	SGA	918	55.4	5.5	38.0	52.0	55.2	59.5	73.5
	No SGA	8071	55.6	5.4	33.2	52.2	55.5	59.2	76.9
	FGR	587	55.5	5.5	38.0	51.9	55.2	59.5	73.5
	No FGR	8407	55.6	5.4	33.2	52.2	55.5	59.2	76.9
- $L_{Aeq,day}$	All	8994	56.7	5.2	34.3	53.4	56.7	60.3	76.4
	SGA	918	56.6	5.4	39.0	53.2	56.5	60.7	72.7
	No SGA	8071	56.7	5.2	34.3	53.4	56.8	60.3	76.4
	FGR	587	56.6	5.3	39.0	53.2	56.5	60.5	72.7
	No FGR	8407	56.7	5.2	34.3	53.4	56.8	60.3	76.4
- $L_{Aeq,evening}$	All	8994	56.0	5.8	34.8	52.2	56.0	59.9	77.8
	SGA	918	55.6	6.0	36.8	51.7	55.4	60.0	74.9
	No SGA	8071	56.0	5.8	34.8	52.3	56.1	59.9	77.8
	FGR	587	55.8	5.9	36.8	51.9	55.8	59.7	74.9
	No FGR	8407	56.0	5.8	34.8	52.2	56.1	59.9	77.8
- $L_{Aeq,night}$	All	8994	51.9	5.8	28.2	48.1	51.8	55.7	77.0
	SGA	918	51.8	6.0	33.3	47.7	51.8	55.5	73.8
	No SGA	8071	52.0	5.8	28.2	48.2	51.8	55.7	77.0
	FGR	587	51.9	5.9	33.3	47.9	51.7	55.8	73.8
	No FGR	8407	52.0	5.8	28.2	48.1	51.8	55.7	77.0
- L_{den}	All	8994	58.1	6.3	34.2	54.0	57.8	62.3	83.5
	SGA	918	57.8	6.4	39.1	53.6	57.6	62.2	80.2
	No SGA	8071	58.1	6.3	34.2	54.0	57.8	62.3	83.5
	FGR	587	57.9	6.3	39.1	53.9	57.7	62.1	80.2
	No FGR	8407	58.1	6.3	34.2	54.0	57.8	62.3	83.5
Road traffic related noise exposure (dB) building									
- $L_{Aeq,24h}$	All	8994	54.4	5.2	33.2	51.1	54.6	58.0	69.6
	SGA	918	54.3	5.4	35.2	50.8	54.4	58.3	69.1
	No SGA	8071	54.4	5.2	33.2	51.2	54.6	57.9	69.6
	FGR	587	54.2	5.4	34.8	51.1	54.4	58.1	69.3
	No FGR	8407	54.4	5.2	33.2	51.2	54.6	58.0	69.6
Rail traffic related noise exposure (dB) building									
- $L_{Aeq,24h}$	All	8994	25.0	21.3	0.0	0.0	28.5	44.0	72.4
	SGA	918	24.7	20.8	0.0	0.0	28.4	42.2	70.4
	No SGA	8071	25.1	21.4	0.0	0.0	28.6	44.2	72.4
	FGR	587	25.3	21.1	0.0	0.0	28.2	44.2	70.4
	No FGR	8407	25.0	21.4	0.0	0.0	28.5	44.0	72.4
NO_2 concentration, 50m radius buffer (µg/m^3)									
- first trimester	All	8994	24.6	7.8	5.9	18.8	23.3	29.7	64.1
	SGA	918	24.3	8.0	8.2	18.3	23.0	29.3	52.6
	No SGA	8071	24.7	7.8	5.9	18.9	23.3	29.7	64.1
	FGR	587	24.2	7.7	6.8	18.7	23.0	29.2	46.3
	No FGR	8407	24.7	7.8	5.9	18.8	23.3	29.7	64.1
- second trimester	All	8994	24.4	7.7	6.0	18.7	23.1	29.4	56.3
	SGA	918	24.2	8.0	6.9	18.3	22.5	28.9	52.5

	No SGA	8071	24.5	7.7	6.0	18.7	23.2	29.4	56.3
	FGR	587	24.4	7.8	6.9	18.8	23.1	29.1	55.6
	No FGR	8407	24.4	7.7	6.0	18.7	23.1	29.4	56.3
- third trimester	All	8887	24.4	7.7	6.9	18.7	23.1	29.5	54.9
	SGA	893	24.2	7.9	7.5	18.2	22.8	29.0	50.8
	No SGA	7990	24.5	7.7	6.9	18.8	23.2	29.5	54.9
	FGR	572	24.6	7.7	9.0	18.9	23.5	29.6	50.8
	No FGR	8315	24.4	7.7	6.9	18.7	23.1	29.5	54.9
- entire pregnancy	All	8994	24.5	7.4	7.4	19.0	23.2	29.3	51.6
	SGA	918	24.2	7.6	8.8	18.6	22.9	29.0	47.1
	No SGA	8071	24.5	7.4	7.4	19.1	23.2	29.3	51.6
	FGR	587	24.4	7.4	8.4	19.0	23.2	29.1	50.4
	No FGR	8407	24.5	7.4	7.4	19.0	23.2	29.3	51.6
- the two months before delivery	All	8994	24.5	7.8	6.7	18.7	23.1	29.5	57.1
	SGA	918	24.2	8.0	7.1	18.3	22.7	28.9	53.3
	No SGA	8071	24.5	7.8	6.7	18.7	23.1	29.5	57.1
	FGR	587	24.7	7.8	9.5	18.7	23.2	29.7	57.1
	No FGR	8407	24.4	7.8	6.7	18.7	23.1	29.5	54.9
PM_{10} concentration, 50m radius buffer (µg/m^3)									
- first trimester	All	8994	18.7	3.9	10.9	15.8	18.1	20.8	35.2
	SGA	918	18.7	3.9	11.4	15.8	18.1	21.0	34.2
	No SGA	8071	18.7	3.9	10.9	15.8	18.1	20.8	35.2
	FGR	587	18.4	3.8	11.9	15.6	17.7	20.4	33.3
	No FGR	8407	18.7	3.9	10.9	15.8	18.1	20.9	35.2
- second trimester	All	8994	18.6	3.9	9.8	15.8	18.1	20.8	35.6
	SGA	918	18.8	3.9	10.3	16.0	18.3	21.2	31.9
	No SGA	8071	18.6	3.9	9.8	15.7	18.1	20.8	35.6
	FGR	587	18.6	3.8	10.5	16.0	18.3	20.6	31.9
	No FGR	8407	18.6	3.9	9.8	15.7	18.1	20.8	35.6
- third trimester	All	8887	18.6	4.0	9.4	15.7	18.0	20.8	38.8
	SGA	893	18.9	4.0	10.6	16.0	18.3	21.2	33.2
	No SGA	7990	18.6	4.0	9.4	15.7	18.0	20.7	38.8
	FGR	572	19.1	4.3	10.6	16.1	18.5	21.4	33.2
	No FGR	8315	18.5	4.0	9.4	15.7	18.0	20.7	38.8
- entire pregnancy	All	8994	18.6	2.7	11.9	16.7	18.5	20.3	31.5
	SGA	918	18.8	2.6	12.2	16.9	18.8	20.6	27.3
	No SGA	8071	18.6	2.7	11.9	16.7	18.4	20.3	31.5
	FGR	587	18.7	2.7	12.6	16.7	18.6	20.4	26.6
	No FGR	8407	18.6	2.7	11.9	16.7	18.5	20.3	31.5
- the two months before delivery	All	8994	18.6	4.2	9.5	15.6	18.0	20.8	38.3
	SGA	918	18.9	4.1	10.5	15.8	18.4	21.2	33.2
	No SGA	8071	18.5	4.2	9.5	15.6	17.9	20.8	38.3
	FGR	587	19.1	4.4	10.4	15.9	18.6	21.4	33.2
	No FGR	8407	18.5	4.2	9.5	15.6	18.0	20.8	38.3

N: number; SD: standard deviation; Q1: first quartile; Q3: third quartile; FGR: fetal growth restriction; SGA: small for gestational age

Table S3. Pearson's correlation coefficients between environmental exposures for all pregnancies (N = 8994)

	Total noise exposure (dB) building					Road traffic related noise exposure (dB) building $L_{Aeq,24h}$	Rail traffic related noise exposure (dB) building $L_{Aeq,24h}$	NO_2 concentration, 50m radius buffer (µg/m^3)					PM_{10} concentration, 50m radius buffer (µg/m^3)				
	$L_{Aeq,24h}$	$L_{Aeq,day}$	$L_{Aeq,evening}$	$L_{Aeq,night}$	L_{den}			first trimester	second trimester	third trimester	entire pregnancy	the two months before delivery	first trimester	second trimester	third trimester	entire pregnancy	the two months before delivery
Total noise exposure (dB) building																	
- $L_{Aeq,24h}$	1.00	0.99	0.98	0.96	0.96	0.88	0.37	0.53	0.53	0.53	0.56	0.53	0.19	0.20	0.18	0.28	0.18
- $L_{Aeq,day}$		1.00	0.96	0.94	0.93	0.91	0.32	0.51	0.52	0.52	0.54	0.51	0.20	0.21	0.20	0.30	0.19
- $L_{Aeq,evening}$			1.00	0.93	0.96	0.84	0.37	0.58	0.57	0.57	0.60	0.57	0.15	0.15	0.13	0.21	0.12
- $L_{Aeq,night}$				1.00	0.97	0.77	0.50	0.49	0.49	0.49	0.52	0.49	0.18	0.20	0.18	0.28	0.17
- L_{den}					1.00	0.76	0.56	0.56	0.56	0.56	0.59	0.56	0.16	0.17	0.14	0.23	0.13
Road traffic related noise exposure (dB) building $L_{Aeq,24h}$						1.00	0.11	0.48	0.49	0.49	0.51	0.49	0.20	0.21	0.20	0.30	0.20
Rail traffic related noise exposure (dB) building $L_{Aeq,24h}$							1.00	0.26	0.26	0.26	0.28	0.26	0.08	0.09	0.07	0.12	0.06
NO_2 concentration, 50m radius buffer (µg/m^3)																	
- first trimester								1.00	0.89	0.83	0.95	0.82	0.39	0.20	0.04	0.32	0.04
- second trimester									1.00	0.89	0.97	0.87	0.17	0.38	0.19	0.37	0.16
- third trimester										1.00	0.94	0.99	0.04	0.17	0.35	0.27	0.34
- entire pregnancy											1.00	0.93	0.22	0.27	0.19	0.34	0.18
- the two months before delivery												1.00	0.04	0.14	0.35	0.25	0.35
PM_{10} concentration, 50m radius buffer (µg/m^3)																	
- first trimester													1.00	0.31	-0.13	0.62	-0.12
- second trimester														1.00	0.35	0.83	0.27
- third trimester															1.00	0.56	0.97
- entire pregnancy																1.00	0.51
- the two months before delivery																	1.00

Table S4. Environmental exposures according to the city of residence, 2005–2009 (N = 8994)

	N	*Total N (%) or μ (SD)*	Besançon N (%) or μ (SD)	Dijon N (%) or μ (SD)	P-value [a]
Total noise exposure (dB) building					
- $L_{Aeq,24h}$	8994	*55.6 (5.4)*	53.9 (5.4)	56.8 (5.0)	$< 10^{-3}$
- $L_{Aeq,day}$	8994	*56.7 (5.2)*	55.4 (5.4)	57.6 (4.9)	$< 10^{-3}$
- $L_{Aeq,evening}$	8994	*56.0 (5.8)*	52.8 (5.5)	58.1 (5.0)	$< 10^{-3}$
- $L_{Aeq,night}$	8994	*51.9 (5.8)*	50.4 (5.6)	53.0 (5.8)	$< 10^{-3}$
- L_{den}	8994	*58.1 (6.3)*	55.2 (5.5)	60.1 (6.0)	$< 10^{-3}$
NO_2 concentration, 50m radius buffer (μg/m^3)					
- first trimester	8994	*24.6 (7.8)*	20.6 (6.3)	27.4 (7.6)	$< 10^{-3}$
- second trimester	8994	*24.4 (7.7)*	20.7 (6.2)	27.0 (7.6)	$< 10^{-3}$
- third trimester	8887	*24.4 (7.7)*	20.8 (6.1)	27.0 (7.7)	$< 10^{-3}$
- entire pregnancy	8994	*24.5 (7.4)*	20.7 (5.7)	27.1 (7.3)	$< 10^{-3}$
- the two months before delivery	8994	*24.5 (7.8)*	20.8 (6.2)	27.0 (7.8)	$< 10^{-3}$
PM_{10} concentration, 50m radius buffer (μg/m^3)					
- first trimester	8994	*18.7 (3.9)*	19.3 (4.6)	18.3 (3.3)	$< 10^{-3}$
- second trimester	8994	*18.6 (3.9)*	19.4 (4.4)	18.0 (3.4)	$< 10^{-3}$
- third trimester	8887	*18.6 (4.0)*	19.6 (4.5)	17.9 (3.5)	$< 10^{-3}$
- entire pregnancy	8994	*18.6 (2.7)*	19.4 (2.9)	18.1 (2.3)	$< 10^{-3}$
- the two months before delivery	8994	*18.6 (4.2)*	19.6 (4.7)	17.9 (3.6)	$< 10^{-3}$

N: number; N (%): number (percentage), μ: mean, SD: standard deviation.
[a] P-value of Student t-test.

Table S5. Sensitivity analyses on noise exposure sources. Relationship between total noise, road traffic related noise, rail traffic related noise, NO_2 and PM_{10} exposure during pregnancy and small for gestational age or fetal growth restriction, 2005-2009 (N = 8994)

Single-exposure models	Outcome		OR [95% CI] for an increase of 5 dB or 10 µg/m^3			
	Yes *µ (SD)*	No *µ (SD)*	Crude OR	p-value [a]	Adjusted OR [b]	p-value [a]
**Small for gestational age**	N = 918	N = 8071	N = 8989		N = 8989	
Noise exposure (dB) building $L_{Aeq,24h}$						
- total noise	55.4 (5.5)	55.6 (5.4)	0.97 [0.91; 1.03]	0.30	0.97 [0.91; 1.03]	0.31
- road traffic related noise	54.3 (5.4)	54.4 (5.2)	0.98 [0.92; 1.05]	0.58	0.99 [0.92; 1.06]	0.68
- rail traffic related noise	24.7 (20.8)	25.1 (21.4)	1.00 [0.98; 1.01]	0.57	0.99 [0.98; 1.01]	0.40
**Fetal growth restriction**	N = 587	N = 8407	N = 8994		N = 8994	
Noise exposure (dB) building $L_{Aeq,24h}$						
- total noise	55.5 (5.5)	55.6 (5.4)	0.98 [0.90; 1.06]	0.55	0.98 [0.90; 1.06]	0.58
- road traffic related noise	54.2 (5.4)	54.4 (5.2)	0.97 [0.90; 1.05]	0.47	0.98 [0.90; 1.06]	0.61
- rail traffic related noise	25.3 (21.1)	25.0 (21.4)	1.00 [0.98; 1.02]	0.75	1.00 [0.98; 1.02]	0.99

Two-exposure models	OR [95% CI] for an increase of 5 dB or 10 µg/m^3					
	Total noise		Road traffic related noise		Rail traffic related noise	
	Adjusted OR [b]	p-value [a]	Adjusted OR [b]	p-value [a]	Adjusted OR [b]	p-value [a]
**Small for gestational age**	N = 8989		N = 8989		N = 8989	
Noise exposure (dB) building $L_{Aeq,24h}$ [c]						
- adjusted on NO_2	1.00 [0.92; 1.08]	0.89	1.02 [0.94; 1.10]	0.64	1.00 [0.98; 1.01]	0.68
- adjusted on PM_{10}	0.95 [0.89; 1.02]	0.15	0.97 [0.90; 1.04]	0.40	0.99 [0.98; 1.01]	0.31
NO_2 concentration, 50m radius buffer (µg/m^3)						
- first trimester	0.93 [0.83; 1.03]	0.16	0.91 [0.82; 1.01]	0.08	0.93 [0.84; 1.02]	0.12
- second trimester	0.94 [0.85; 1.05]	0.27	0.93 [0.83; 1.03]	0.15	0.94 [0.85; 1.03]	0.20
- third trimester [d]	0.93 [0.84; 1.04]	0.22	0.92 [0.83; 1.02]	0.12	0.93 [0.85; 1.03]	0.15
- entire pregnancy	0.93 [0.83; 1.04]	0.18	0.91 [0.81; 1.02]	0.09	0.93 [0.84; 1.02]	0.14
- the two months before delivery	0.95 [0.86; 1.06]	0.36	0.94 [0.84; 1.04]	0.21	0.95 [0.86; 1.04]	0.26

PM_{10} concentration, 50m radius buffer (µg/m^3)						
- first trimester	1.02 [0.85; 1.23]	0.81	1.01 [0.84; 1.21]	0.90	1.01 [0.85; 1.21]	0.91
- second trimester	1.13 [0.95; 1.36]	0.18	1.12 [0.94; 1.35]	0.21	1.12 [0.93; 1.33]	0.23
- third trimester [d]	1.19 [1.00; 1.41]	0.06	1.18 [0.99; 1.40]	0.07	1.17 [0.98; 1.39]	0.08
- entire pregnancy	1.25 [0.95; 1.64]	0.11	1.22 [0.93; 1.61]	0.15	1.20 [0.92; 1.56]	0.17
- the two months before delivery	1.18 [1.00; 1.40]	0.05	1.18 [0.99; 1.39]	0.06	1.17 [0.99; 1.38]	0.07
**Fetal growth restriction**	N = 8994		N = 8994		N = 8994	
Noise exposure (dB) building $L_{Aeq,24h}$ [c]						
- adjusted on NO_2	0.99 [0.90; 1.09]	0.80	0.99 [0.90; 1.09]	0.81	1.00 [0.98; 1.02]	0.86
- adjusted on PM_{10}	0.98 [0.90; 1.06]	0.61	0.98 [0.90; 1.07]	0.63	1.00 [0.98; 1.02]	0.97
NO_2 concentration, 50m radius buffer (µg/m^3)						
- first trimester	0.91 [0.80; 1.04]	0.15	0.91 [0.80; 1.04]	0.15	0.91 [0.81; 1.02]	0.12
- second trimester	0.99 [0.87; 1.13]	0.91	0.99 [0.89; 1.08]	0.88	0.98 [0.87; 1.10]	0.70
- third trimester [e]	1.04 [0.91; 1.19]	0.54	1.03 [0.91; 1.17]	0.65	1.02 [0.90; 1.14]	0.78
- entire pregnancy	0.97 [0.85; 1.12]	0.70	0.97 [0.85; 1.11]	0.67	0.96 [0.85; 1.09]	0.52
- the two months before delivery	1.06 [0.93; 1.20]	0.41	1.05 [0.93; 1.19]	0.44	1.03 [0.92; 1.15]	0.65
PM_{10} concentration, 50m radius buffer (µg/m^3)						
- first trimester	0.77 [0.61; 0.97]	0.03	0.77 [0.61; 0.97]	0.03	0.77 [0.61; 0.97]	0.02
- second trimester	0.97 [0.77; 1.22]	0.78	0.97 [0.77; 1.22]	0.78	0.96 [0.77; 1.20]	0.70
- third trimester [e]	1.38 [1.12; 1.70]	< 0.01	1.38 [1.12; 1.70]	< 0.01	1.35 [1.10; 1.67]	< 0.01
- entire pregnancy	0.99 [0.71; 1.39]	0.97	0.99 [0.71; 1.39]	0.97	0.97 [0.70; 1.34]	0.84
- the two months before delivery	1.35 [1.11; 1.66]	< 0.01	1.36 [1.11; 1.67]	< 0.01	1.33 [1.09; 1.63]	< 0.01

N: number; µ (SD): exposure average (standard deviation); OR: Odds ratio; CI: confidence interval.
[a] Wald Chi-square test.
[b] adjusted for term, maternal age above 35 years at delivery, low neighborhood socioeconomic level, maternal smoking during pregnancy, malnutrition and obesity, nulliparity, gestational hypertension, diabetes, assisted reproductive techniques, vaginal bleeding in the second and third trimesters, infection, major infant congenital abnormalities.
[c] entire pregnancy for NO_2 and PM_{10} indices in adjusted analyses (the results were similar with the other period indices).
[d] missing data for delivery before 29 weeks of gestational age (n = 106).
[e] missing data for delivery before 29 weeks of gestational age (n = 107).

Table S6. Sensitivity analyses on adjustment for maternal age at delivery. Relationship between total noise, NO_2 and PM_{10} exposure during pregnancy and small for gestational age or fetal growth restriction, with maternal age at delivery considered for adjustment in continuous form, or with a second or third order polynomial, 2005-2009 (N = 8994)

Two-exposure models	OR [95% CI] for an increase of 5 dB or 10 µg/m³ With maternal age at delivery:							
	Above 35 years		In continuous form		With a second order polynomial		With a third order polynomial	
	Adjusted OR [b]	p-value [a]	Adjusted OR [b]	p-value [a]	Adjusted OR [b]	p-value [a]	Adjusted OR [b]	p-value [a]
**Small for gestational age**	N = 8989		N = 8989		N = 8989		N = 8989	
Noise exposure (dB) building $L_{Aeq,24h}$ [c]								
- adjusted on NO_2	1.00 [0.92; 1.08]	0.89	1.00 [0.92; 1.08]	0.90	1.00 [0.92; 1.08]	0.90	1.00 [0.92; 1.08]	0.91
- adjusted on PM_{10}	0.95 [0.89; 1.02]	0.15	0.95 [0.89; 1.02]	0.16	0.95 [0.89; 1.02]	0.16	0.95 [0.89; 1.02]	0.16
NO_2 concentration, 50m radius buffer (µg/m³)								
- first trimester	0.93 [0.83; 1.03]	0.16	0.93 [0.83; 1.03]	0.17	0.93 [0.83; 1.03]	0.17	0.93 [0.83; 1.03]	0.17
- second trimester	0.94 [0.85; 1.05]	0.27	0.94 [0.85; 1.05]	0.28	0.94 [0.85; 1.05]	0.28	0.94 [0.85; 1.05]	0.28
- third trimester [d]	0.93 [0.84; 1.04]	0.22	0.94 [0.84; 1.04]	0.22	0.94 [0.84; 1.04]	0.23	0.94 [0.84; 1.04]	0.23
- entire pregnancy	0.93 [0.83; 1.04]	0.18	0.93 [0.83; 1.04]	0.19	0.93 [0.83; 1.04]	0.19	0.93 [0.83; 1.04]	0.19
- the two months before delivery	0.95 [0.86; 1.06]	0.36	0.95 [0.86; 1.06]	0.37	0.95 [0.86; 1.06]	0.37	0.95 [0.86; 1.06]	0.37
PM_{10} concentration, 50m radius buffer (µg/m³)								
- first trimester	1.02 [0.85; 1.23]	0.81	1.02 [0.85; 1.23]	0.81	1.02 [0.85; 1.23]	0.81	1.02 [0.85; 1.23]	0.81
- second trimester	1.13 [0.95; 1.36]	0.18	1.13 [0.95; 1.36]	0.18	1.13 [0.94; 1.36]	0.18	1.13 [0.95; 1.36]	0.18
- third trimester [d]	1.19 [1.00; 1.41]	0.06	1.19 [1.00; 1.41]	0.06	1.19 [1.00; 1.41]	0.06	1.19 [1.00; 1.41]	0.06
- entire pregnancy	1.25 [0.95; 1.64]	0.11	1.25 [0.95; 1.64]	0.11	1.25 [0.95; 1.64]	0.11	1.25 [0.95; 1.64]	0.11
- the two months before delivery	1.18 [1.00; 1.40]	0.05	1.18 [1.00; 1.40]	0.05	1.18 [1.00; 1.40]	0.05	1.18 [1.00; 1.40]	0.05
**Fetal growth restriction**	N = 8994		N = 8994		N = 8994		N = 8994	
Noise exposure (dB) building $L_{Aeq,24h}$ [c]								
- adjusted on NO_2	0.99 [0.90; 1.09]	0.80	0.99 [0.90; 1.09]	0.84	0.99 [0.90; 1.09]	0.84	0.99 [0.90; 1.09]	0.84
- adjusted on PM_{10}	0.98 [0.90; 1.06]	0.61	0.98 [0.90; 1.07]	0.67	0.98 [0.90; 1.07]	0.68	0.98 [0.90; 1.07]	0.68
NO_2 concentration, 50m radius buffer (µg/m³)								
- first trimester	0.91 [0.80; 1.04]	0.15	0.91 [0.80; 1.04]	0.17	0.91 [0.80; 1.04]	0.17	0.91 [0.80; 1.04]	0.17
- second trimester	0.99 [0.87; 1.13]	0.91	1.00 [0.87; 1.14]	0.96	1.00 [0.87; 1.14]	0.96	1.00 [0.87; 1.14]	0.96
- third trimester [e]	1.04 [0.91; 1.19]	0.54	1.04 [0.92; 1.19]	0.52	1.05 [0.92; 1.19]	0.51	1.05 [0.92; 1.19]	0.51

- entire pregnancy	0.97 [0.85; 1.12]	0.70	0.98 [0.85; 1.12]	0.74	0.98 [0.85; 1.12]	0.74	0.98 [0.85; 1.12]	0.74
- the two months before delivery	1.06 [0.93; 1.20]	0.41	1.06 [0.93; 1.21]	0.38	1.06 [0.93; 1.21]	0.38	1.06 [0.93; 1.21]	0.38
PM_{10} concentration, 50m radius buffer (µg/m^3)								
- first trimester	0.77 [0.61; 0.97]	0.03	0.77 [0.61; 0.97]	0.03	0.77 [0.61; 0.97]	0.03	0.77 [0.61; 0.97]	0.03
- second trimester	0.97 [0.77; 1.22]	0.78	0.97 [0.77; 1.22]	0.79	0.97 [0.77; 1.22]	0.77	0.97 [0.77; 1.22]	0.77
- third trimester [e]	1.38 [1.12; 1.70]	< 0.01	1.37 [1.11; 1.70]	< 0.01	1.38 [1.11; 1.70]	< 0.01	1.38 [1.11; 1.70]	< 0.01
- entire pregnancy	0.99 [0.71; 1.39]	0.97	0.99 [0.71; 1.38]	0.96	0.99 [0.71; 1.38]	0.95	0.99 [0.71; 1.38]	0.95
- the two months before delivery	1.35 [1.11; 1.66]	< 0.01	1.35 [1.10; 1.66]	< 0.01	1.35 [1.10; 1.66]	< 0.01	1.35 [1.10; 1.66]	< 0.01

N: number; µ (SD): exposure average (standard deviation); OR: Odds ratio; CI: confidence interval.

[a] Wald Chi-square test.

[b] adjusted for term, maternal age considered in different forms, low neighborhood socioeconomic level, maternal smoking during pregnancy, malnutrition and obesity, nulliparity, gestational hypertension, diabetes, assisted reproductive techniques, vaginal bleeding in the second and third trimesters, infection, major infant congenital abnormalities.

[c] entire pregnancy for NO_2 and PM_{10} indices in adjusted analyses (the results were similar with the other period indices).

[d] missing data for delivery before 29 weeks of gestational age (n = 106).

[e] missing data for delivery before 29 weeks of gestational age (n = 107).

Table S7. Sensitivity analyses on adjustment for neighborhood socioeconomic level. Relationship between total noise, NO_2 and PM_{10} exposure during pregnancy and small for gestational age or fetal growth restriction, with neighborhood socioeconomic level considered for adjustment in continuous form, or with a second or third order polynomial, 2005-2009 (N = 8994)

Two-exposure models	OR [95% CI] for an increase of 5 dB or 10 µg/m^3 With neighborhood socioeconomic level:							
	In the last decile		In continuous form		With a second order polynomial		With a third order polynomial	
	Adjusted OR [b]	p-value [a]	Adjusted OR [b]	p-value [a]	Adjusted OR [b]	p-value [a]	Adjusted OR [b]	p-value [a]
**Small for gestational age**	N = 8989		N = 8989		N = 8989		N = 8989	
Noise exposure (dB) building $L_{Aeq,24h}$ [c]								
- adjusted on NO_2	1.00 [0.92; 1.08]	0.89	1.00 [0.93; 1.08]	0.99	1.00 [0.93; 1.08]	0.99	1.00 [0.93; 1.08]	0.99
- adjusted on PM_{10}	0.95 [0.89; 1.02]	0.15	0.95 [0.89; 1.02]	0.14	0.95 [0.89; 1.02]	0.17	0.95 [0.89; 1.02]	0.17
NO_2 concentration, 50m radius buffer (µg/m^3)								
- first trimester	0.93 [0.83; 1.03]	0.16	0.91 [0.81; 1.01]	0.08	0.91 [0.82; 1.02]	0.10	0.91 [0.82; 1.02]	0.10
- second trimester	0.94 [0.85; 1.05]	0.27	0.92 [0.83; 1.03]	0.14	0.93 [0.83; 1.03]	0.17	0.93 [0.83; 1.03]	0.17
- third trimester [d]	0.93 [0.84; 1.04]	0.22	0.91 [0.82; 1.02]	0.11	0.92 [0.82; 1.03]	0.13	0.92 [0.82; 1.03]	0.14
- entire pregnancy	0.93 [0.83; 1.04]	0.18	0.90 [0.80; 1.02]	0.09	0.91 [0.81; 1.02]	0.11	0.91 [0.81; 1.02]	0.11
- the two months before delivery	0.95 [0.86; 1.06]	0.36	0.93 [0.84; 1.04]	0.21	0.94 [0.84; 1.05]	0.24	0.94 [0.84; 1.05]	0.25
PM_{10} concentration, 50m radius buffer (µg/m^3)								
- first trimester	1.02 [0.85; 1.23]	0.81	1.02 [0.85; 1.22]	0.86	1.02 [0.85; 1.22]	0.83	1.02 [0.85; 1.23]	0.82
- second trimester	1.13 [0.95; 1.36]	0.18	1.13 [0.94; 1.35]	0.20	1.13 [0.94; 1.35]	0.19	1.13 [0.94; 1.36]	0.18
- third trimester [d]	1.19 [1.00; 1.41]	0.06	1.18 [0.99; 1.41]	0.06	1.18 [0.99; 1.41]	0.06	1.19 [1.00; 1.41]	0.06
- entire pregnancy	1.25 [0.95; 1.64]	0.11	1.24 [0.94; 1.62]	0.13	1.24 [0.95; 1.63]	0.12	1.25 [0.95; 1.64]	0.11
- the two months before delivery	1.18 [1.00; 1.40]	0.05	1.18 [1.00; 1.39]	0.05	1.18 [1.00; 1.40]	0.05	1.19 [1.00; 1.40]	0.05
**Fetal growth restriction**	N = 8994		N = 8994		N = 8994		N = 8994	
Noise exposure (dB) building $L_{Aeq,24h}$ [c]								
- adjusted on NO_2	0.99 [0.90; 1.09]	0.80	0.99 [0.90; 1.09]	0.83	0.99 [0.90; 1.09]	0.81	0.99 [0.90; 1.09]	0.79
- adjusted on PM_{10}	0.98 [0.90; 1.06]	0.61	0.97 [0.90; 1.06]	0.55	0.97 [0.89; 1.06]	0.49	0.97 [0.89; 1.06]	0.48
NO_2 concentration, 50m radius buffer (µg/m^3)								
- first trimester	0.91 [0.80; 1.04]	0.15	0.89 [0.78; 1.02]	0.10	0.88 [0.77; 1.01]	0.08	0.88 [0.77; 1.01]	0.07
- second trimester	0.99 [0.87; 1.13]	0.91	0.98 [0.86; 1.12]	0.75	0.97 [0.85; 1.11]	0.66	0.97 [0.85; 1.11]	0.65
- third trimester [e]	1.04 [0.91; 1.19]	0.54	1.03 [0.90; 1.18]	0.67	1.02 [0.89; 1.17]	0.74	1.02 [0.89; 1.17]	0.74

- entire pregnancy	0.97 [0.85; 1.12]	0.70	0.96 [0.83; 1.10]	0.54	0.95 [0.82; 1.09]	0.46	0.95 [0.82; 1.09]	0.45
- the two months before delivery	1.06 [0.93; 1.20]	0.41	1.04 [0.92; 1.19]	0.52	1.04 [0.91; 1.18]	0.59	1.04 [0.91; 1.18]	0.60
PM_{10} concentration, 50m radius buffer (µg/m^3)								
- first trimester	0.77 [0.61; 0.97]	0.03	0.77 [0.61; 0.97]	0.03	0.77 [0.60; 0.97]	0.03	0.77 [0.60; 0.96]	0.02
- second trimester	0.97 [0.77; 1.22]	0.78	0.97 [0.77; 1.21]	0.77	0.97 [0.77; 1.21]	0.76	0.96 [0.76; 1.21]	0.73
- third trimester [e]	1.38 [1.12; 1.70]	< 0.01	1.38 [1.12; 1.70]	< 0.01	1.38 [1.12; 1.70]	< 0.01	1.37 [1.11; 1.70]	< 0.01
- entire pregnancy	0.99 [0.71; 1.39]	0.97	0.99 [0.71; 1.38]	0.96	0.98 [0.71; 1.37]	0.92	0.98 [0.70; 1.37]	0.89
- the two months before delivery	1.35 [1.11; 1.66]	< 0.01	1.36 [1.11; 1.66]	< 0.01	1.35 [1.10; 1.66]	< 0.01	1.35 [1.10; 1.66]	< 0.01

N: number; µ (SD): exposure average (standard deviation); OR: Odds ratio; CI: confidence interval.
[a] Wald Chi-square test.
[b] adjusted for term, maternal age above 35 years at delivery, low neighborhood socioeconomic level, maternal smoking during pregnancy, malnutrition and obesity, nulliparity, gestational hypertension, diabetes, assisted reproductive techniques, vaginal bleeding in the second and third trimesters, infection, major infant congenital abnormalities.
[c] entire pregnancy for NO_2 and PM_{10} indices in adjusted analyses (the results were similar with the other period indices).
[d] missing data for delivery before 29 weeks of gestational age (n = 106).
[e] missing data for delivery before 29 weeks of gestational age (n = 107).

Table S8. Sensitivity analysis on live births only. Pregnancy and newborn characteristics according to fetal growth restriction and small for gestational age status, 2005–2009 (N = 8994)

		Total	Small for gestational age [a]		Fetal growth restriction	
			Yes	No	Yes	No
	N	*N (%)*	*N (%)*	*N (%)*	*N (%)*	*N (%)*
**PREGNANCIES**		***N = 8896***	**N = 888**	**N = 8004**	**N = 578**	**N = 8318**
City of residence	8896					
- Besançon		*3647 (41.0)*	419 (47.2)	3224 (40.3)	256 (44.3)	3391 (40.8)
- Dijon		*5249 (59.0)*	469 (52.8)	4780 (59.7)	322 (55.7)	4927 (59.2)
Maternal age at delivery > 35 years old	8896	*1398 (15.7)*	121 (13.6)	1276 (15.9)	80 (13.8)	1318 (15.9)
Low neighborhood socioeconomic level	8896	*1298 (14.6)*	140 (15.8)	1158 (14.5)	82 (14.2)	1216 (14.6)
Living status	8494					
- Living alone		*710 (8.4)*	101 (11.8)	609 (8.0)	65 (11.7)	645 (8.1)
- Married, cohabitation, others		*7784 (91.6)*	755 (88.2)	7025 (92.0)	489 (88.3)	7295 (91.9)
Maternal employment during pregnancy	8421	*5400 (64.1)*	525 (62.1)	4873 (64.4)	346 (62.8)	5054 (64.2)
Maternal smoking during pregnancy	8714	*1649 (18.9)*	313 (35.6)	1336 (17.1)	207 (36.6)	1442 (17.7)
Pre-pregnancy body mass index (kg/m²)	8700					
- < 25		*6179 (71.0)*	681 (78.0)	5494 (70.2)	451 (80.0)	5728 (70.4)
- 25 – 30		*1708 (19.6)*	128 (14.7)	1580 (20.2)	79 (14.0)	1629 (20.0)
- > 30		*813 (9.3)*	64 (7.3)	749 (9.6)	34 (6.0)	779 (9.6)
Malnutrition	8700	*664 (7.6)*	119 (13.6)	545 (7.0)	81 (14.4)	583 (7.2)
Nulliparity	8896	*4760 (53.5)*	573 (64.5)	4184 (52.3)	386 (66.8)	4374 (52.6)
History of medical interruption of pregnancy	8826	*126 (1.4)*	11 (1.2)	115 (1.5)	9 (1.6)	117 (1.4)
History of preterm delivery	8694	*161 (1.9)*	17 (1.9)	144 (1.8)	11 (1.9)	150 (1.9)
Abnormalities of the female reproductive tract	8896	*712 (8.0)*	56 (6.3)	654 (8.2)	47 (8.1)	665 (8.0)
Uterine scar	8896	*608 (6.8)*	45 (5.1)	562 (7.0)	39 (6.8)	569 (6.8)
Assisted reproductive techniques	8896	*185 (2.1)*	19 (2.1)	166 (2.1)	19 (3.3)	166 (2.0)
Gestational hypertension	8896	*377 (4.2)*	72 (8.1)	305 (3.8)	75 (13.0)	302 (3.6)
Vaginal bleeding in the second and third trimesters	8896	*212 (2.4)*	26 (2.9)	186 (2.3)	27 (4.7)	185 (2.2)
Placental abruption	8896	*61(0.7)*	11 (1.2)	50 (0.6)	13 (2.3)	48 (0.6)
Placenta previa	8896	*57 (0.6)*	6 (0.7)	51 (0.6)	4 (0.7)	53 (0.6)
Hemorrhagic placenta previa	8896	*33 (0.4)*	3 (0.3)	30 (0.4)	1 (0.2)	32 (0.4)
Infection	8896	*1240 (13.9)*	128 (14.4)	1112 (13.9)	93 (16.1)	1147 (13.8)
Infection of amniotic fluid	8896	*78 (0.9)*	6 (0.7)	72 (0.9)	10 (1.7)	68 (0.8)
Genitourinary infection	8896	*968 (10.9)*	95 (10.7)	873 (10.9)	58 (10.0)	910 (10.9)
Diabetes	8896	*687 (7.7)*	49 (5.5)	638 (8.0)	33 (5.7)	654 (7.9)
Hydramnios	8896	*113 (1.3)*	5 (0.6)	108 (1.4)	4 (0.7)	109 (1.3)
Premature rupture of membranes	8896	*1166 (13.1)*	88 (9.9)	1077 (13.5)	76 (13.2)	1090 (13.1)
Prematurity (≤ 36 SA)	8896	*654 (7.4)*	67 (7.5)	586 (7.3)	120 (20.8)	534 (6.4)
**NEWBORNS**						
Status	8896					
- Living		*8883 (99.9)*	886 (99.8)	7993 (99.9)	577 (99.8)	8306 (99.9)
- Deceased shortly after birth		*13 (0.1)*	2 (0.2)	11 (0.1)	1 (0.2)	12 (0.1)
Sex	8893					
- Male		*4624 (52.0)*	451 (50.8)	4172 (52.1)	246 (42.7)	4378 (52.6)
- Female		*4269 (48.0)*	437 (49.2)	3832 (47.9)	330 (57.3)	3939 (47.4)
Birth weight (g)	8895	*3236 (557)*	2549 (397)	3313 (517)	2382 (513)	3296 (509)
Major infant congenital abnormalities	8896	*282 (3.2)*	31 (3.5)	249 (3.1)	34 (5.9)	248 (3.0)
Apgar score at five minutes = 10	8794	*8007 (91.1)*	787 (90.2)	7217 (91.2)	485 (86.2)	7522 (91.4)

N: number; N (%): number (percentage) except for birth weight which is described by mean (standard deviation).
[a] lower than 10th centile of birth weight for gestational age.

Table S9. Sensitivity analysis on live births only. Relationship between noise, NO_2 and PM_{10} exposure during pregnancy and small for gestational age or fetal growth restriction, 2005-2009 (N = 8994)

	Outcome		OR [95% CI] for an increase of 5 dB or 10 µg/m³					
			Single-exposure models				Two-exposure models	
	Yes *µ (SD)*	No *µ (SD)*	Crude OR	p-value [a]	Adjusted OR [b]	p-value [a]	Adjusted OR [b]	p-value [a]
**Small for gestational age**	N = 888	N = 8004	N = 8892		N = 8892		N = 8892	
Noise exposure (dB) building $L_{Aeq,24h}$							1.00 [0.92; 1.08] [f]	0.98
- entire pregnancy [c]	55.4 (5.6)	55.6 (5.4)	0.97 [0.91; 1.03]	0.33	0.97 [0.91; 1.04]	0.38	0.96 [0.89; 1.02] [g]	0.19
NO_2 concentration, 50m radius buffer (µg/m³)								
- first trimester	24.3 (8.0)	24.7 (7.8)	0.94 [0.86; 1.02]	0.15	0.92 [0.84; 1.01]	0.09	0.92 [0.83; 1.03]	0.14
- second trimester	24.2 (8.0)	24.5 (7.7)	0.96 [0.88; 1.05]	0.36	0.94 [0.85; 1.03]	0.18	0.94 [0.85; 1.05]	0.30
- third trimester [d]	24.2 (7.9)	24.5 (7.7)	0.95 [0.87; 1.04]	0.27	0.93 [0.85; 1.02]	0.14	0.94 [0.84; 1.05]	0.24
- entire pregnancy	24.2 (7.6)	24.5 (7.4)	0.94 [0.86; 1.04]	0.23	0.92 [0.84; 1.02]	0.11	0.93 [0.82; 1.04]	0.19
- the two months before delivery	24.2 (8.0)	24.5 (7.8)	0.96 [0.88; 1.05]	0.40	0.95 [0.86; 1.04]	0.24	0.95 [0.86; 1.06]	0.40
PM_{10} concentration, 50m radius buffer (µg/m³)								
- first trimester	18.7 (3.9)	18.7 (3.9)	1.02 [0.85; 1.21]	0.86	1.00 [0.83; 1.20]	1.00	1.02 [0.85; 1.22]	0.86
- second trimester	18.8 (3.9)	18.6 (3.9)	1.18 [0.99; 1.40]	0.07	1.11 [0.93; 1.33]	0.25	1.14 [0.95; 1.36]	0.18
- third trimester [d]	18.9 (4.0)	18.5 (4.0)	1.23 [1.04; 1.46]	0.02	1.18 [0.99; 1.40]	0.06	1.20 [1.01; 1.43]	0.04
- entire pregnancy	18.8 (2.6)	18.6 (2.7)	1.29 [1.00; 1.67]	0.05	1.19 [0.91; 1.55]	0.20	1.25 [0.95; 1.65]	0.11
- the two months before delivery	18.9 (4.1)	18.5 (4.2)	1.20 [1.02; 1.41]	0.03	1.16 [0.99; 1.37]	0.08	1.19 [1.00; 1.40]	0.05
**Fetal growth restriction**	N = 578	N = 8318	N = 8896		N = 8896		N = 8896	
Noise exposure (dB) building $L_{Aeq,24h}$							0.98 [0.89; 1.08] [f]	0.71
- entire pregnancy [c]	55.4 (5.5)	55.6 (5.4)	0.97 [0.90; 1.05]	0.48	0.97 [0.89; 1.05]	0.40	0.96 [0.89; 1.05] [g]	0.40
NO_2 concentration, 50m radius buffer (µg/m³)								
- first trimester	24.1 (7.7)	24.6 (7.8)	0.92 [0.82; 1.02]	0.12	0.90 [0.80; 1.01]	0.07	0.90 [0.78; 1.02]	0.10
- second trimester	24.3 (7.8)	24.4 (7.7)	0.98 [0.88; 1.10]	0.74	0.96 [0.85; 1.07]	0.45	0.98 [0.85; 1.11]	0.71
- third trimester [e]	24.5 (7.6)	24.4 (7.7)	1.02 [0.91; 1.14]	0.72	0.99 [0.89; 1.12]	0.91	1.03 [0.90; 1.17]	0.69
- entire pregnancy	24.3 (7.4)	24.5 (7.4)	0.97 [0.86; 1.08]	0.54	0.94 [0.84; 1.06]	0.33	0.96 [0.83; 1.10]	0.53
- the two months before delivery	24.6 (7.7)	24.4 (7.8)	1.02 [0.92; 1.14]	0.67	1.01 [0.90; 1.13]	0.91	1.04 [0.91; 1.19]	0.54
PM_{10} concentration, 50m radius buffer (µg/m³)								
- first trimester	18.4 (3.8)	18.7 (3.9)	0.79 [0.63; 0.98]	0.04	0.77 [0.61; 0.97]	0.03	0.78 [0.61; 0.99]	0.04
- second trimester	18.6 (3.8)	18.6 (3.9)	1.01 [0.81; 1.25]	0.95	0.96 [0.77; 1.20]	0.72	0.98 [0.78; 1.23]	0.85
- third trimester [e]	19.1 (4.3)	18.5 (4.0)	1.40 [1.15; 1.71]	< 0.01	1.36 [1.10; 1.67]	< 0.01	1.39 [1.13; 1.72]	< 0.01
- entire pregnancy	18.7 (2.7)	18.6 (2.7)	1.05 [0.77; 1.44]	0.76	0.99 [0.71; 1.37]	0.94	1.03 [0.73; 1.45]	0.86
- the two months before delivery	19.1 (4.4)	18.5 (4.2)	1.35 [1.11; 1.64]	< 0.01	1.36 [1.11; 1.66]	< 0.01	1.39 [1.13; 1.71]	< 0.01

N: number; µ (SD): exposure average (standard deviation); OR: Odds ratio; CI: confidence interval.
[a] Wald Chi-square test.
[b] adjusted for term, maternal age above 35 years at delivery, low neighborhood socioeconomic level, maternal smoking during pregnancy, malnutrition and obesity, nulliparity, gestational hypertension, diabetes, assisted reproductive techniques, vaginal bleeding in the second and third trimesters, infection, major infant congenital abnormalities.
[c] for NO_2 and PM_{10} indices in adjusted analyses (the results were similar with the other period indices).
[d] missing data for delivery before 29 weeks of gestational age (n = 50).
[e] missing data for delivery before 29 weeks of gestational age (n = 51).
[f] noise and NO_2
[g] noise and PM_{10}

Table S10. Sensitivity analysis with adjustment for year-season of conception. Relationship between noise, NO_2 and PM_{10} exposure during pregnancy and small for gestational age or fetal growth restriction, 2005-2009 (N = 8994)

	Outcome		OR [95% CI] for an increase of 5 dB or 10 µg/m³					
			Single-exposure models				Two-exposure models	
	Yes *µ (SD)*	No *µ (SD)*	Crude OR	p-value [a]	Adjusted OR [b]	p-value [a]	Adjusted OR [b]	p-value [a]
**Small for gestational age**	N = 918	N = 8071	N = 8989		N = 8989		N = 8989	
Total noise exposure (dB) building $L_{Aeq,24h}$							1.00 [0.92; 1.08] [f]	0.89
- entire pregnancy [c]	55.4 (5.5)	55.6 (5.4)	0.97 [0.91; 1.03]	0.30	0.97 [0.91; 1.03]	0.31	0.95 [0.89; 1.02] [g]	0.13
NO_2 concentration, 50m radius buffer (µg/m³)								
- first trimester	24.3 (8.0)	24.7 (7.8)	0.94 [0.86; 1.02]	0.15	0.92 [0.84; 1.01]	0.09	0.93 [0.92; 1.07]	0.17
- second trimester	24.2 (8.0)	24.5 (7.7)	0.96 [0.87; 1.04]	0.31	0.93 [0.85; 1.02]	0.14	0.94 [0.85; 1.05]	0.27
- third trimester [d]	24.2 (7.9)	24.5 (7.7)	0.95 [0.87; 1.04]	0.25	0.93 [0.85; 1.02]	0.13	0.93 [0.84; 1.04]	0.22
- entire pregnancy	24.2 (7.6)	24.5 (7.4)	0.94 [0.86; 1.03]	0.20	0.92 [0.84; 1.02]	0.10	0.93 [0.83; 1.04]	0.18
- the two months before delivery	24.2 (8.0)	24.5 (7.8)	0.96 [0.88; 1.05]	0.34	0.94 [0.86; 1.03]	0.19	0.95 [0.86; 1.06]	0.35
PM_{10} concentration, 50m radius buffer (µg/m³)								
- first trimester	18.7 (3.9)	18.7 (3.9)	1.02 [0.86; 1.22]	0.80	1.02 [0.84; 1.23]	0.88	1.04 [0.85; 1.26]	0.72
- second trimester	18.8 (3.9)	18.6 (3.9)	1.17 [0.99; 1.39]	0.07	1.12 [0.93; 1.34]	0.23	1.15 [0.95; 1.38]	0.15
- third trimester [d]	18.9 (4.0)	18.6 (4.0)	1.21 [1.03; 1.43]	0.02	1.17 [0.98; 1.39]	0.08	1.19 [1.00; 1.42]	0.05
- entire pregnancy	18.8 (2.6)	18.6 (2.7)	1.29 [1.00; 1.66]	0.05	1.22 [0.93; 1.61]	0.15	1.31 [0.98; 1.75]	0.07
- the two months before delivery	18.9 (4.1)	18.5 (4.2)	1.20 [1.02; 1.41]	0.03	1.17 [0.99; 1.37]	0.07	1.19 [1.01; 1.41]	0.04
**Fetal growth restriction**	N = 587	N = 8407	N = 8994		N = 8994		N = 8994	
Total noise exposure (dB) building $L_{Aeq,24h}$							0.99 [0.90; 1.09] [f]	0.82
- entire pregnancy [c]	55.5 (5.5)	55.6 (5.4)	0.98 [0.90; 1.06]	0.55	0.98 [0.90; 1.06]	0.60	0.97 [0.89; 1.05] [g]	0.45
NO_2 concentration, 50m radius buffer (µg/m³)								
- first trimester	24.2 (7.7)	24.7 (7.8)	0.93 [0.83; 1.03]	0.18	0.92 [0.83; 1.04]	0.17	0.92 [0.80; 1.05]	0.20
- second trimester	24.4 (7.8)	24.4 (7.7)	1.00 [0.90; 1.11]	0.97	0.98 [0.87; 1.09]	0.68	0.99 [0.87; 1.13]	0.86
- third trimester [e]	24.6 (7.7)	24.4 (7.7)	1.03 [0.93; 1.15]	0.56	1.00 [0.89; 1.12]	0.97	1.04 [0.91; 1.18]	0.59
- entire pregnancy	24.4 (7.4)	24.5 (7.4)	0.98 [0.88; 1.10]	0.73	0.96 [0.86; 1.09]	0.55	0.97 [0.85; 1.12]	0.70
- the two months before delivery	24.7 (7.8)	24.4 (7.8)	1.04 [0.93; 1.16]	0.49	1.02 [0.91; 1.14]	0.70	1.05 [0.92; 1.20]	0.46
PM_{10} concentration, 50m radius buffer (µg/m³)								
- first trimester	18.4 (3.8)	18.7 (3.9)	0.78 [0.63; 0.98]	0.03	0.83 [0.65; 1.06]	0.13	0.83 [0.65; 1.07]	0.15
- second trimester	18.6 (3.8)	18.6 (3.9)	1.02 [0.82; 1.26]	0.87	1.01 [0.80; 1.27]	0.92	1.03 [0.81; 1.30]	0.83
- third trimester [e]	19.1 (4.3)	18.5 (4.0)	1.39 [1.14; 1.70]	< 0.01	1.40 [1.13; 1.73]	< 0.01	1.43 [1.16; 1.78]	< 0.01
- entire pregnancy	18.7 (2.7)	18.6 (2.7)	1.05 [0.77; 1.43]	0.77	1.12 [0.80; 1.57]	0.51	1.17 [0.82; 1.67]	0.39
- the two months before delivery	19.1 (4.4)	18.5 (4.2)	1.35 [1.11; 1.63]	< 0.01	1.38 [1.12; 1.69]	< 0.01	1.41 [1.14; 1.73]	< 0.01

N: number; µ (SD): exposure average (standard deviation); OR: Odds ratio; CI: confidence interval.
[a] Wald Chi-square test.
[b] adjusted for term, year-season of conception, maternal age above 35 years at delivery, low neighborhood socioeconomic level, maternal smoking during pregnancy, malnutrition and obesity, nulliparity, gestational hypertension, diabetes, assisted reproductive techniques, vaginal bleeding in the second and third trimesters, infection, major infant congenital abnormalities.
[c] for NO_2 and PM_{10} indices in adjusted analyses (the results were similar with the other period indices).
[d] missing data for delivery before 29 weeks of gestational age (n = 106).
[e] missing data for delivery before 29 weeks of gestational age (n = 107).
[f] noise and NO_2.
[g] noise and PM_{10}

Figure S1. NO_2 exposure, considering a 50m radius buffer, during different periods of pregnancy, according to fetal growth restriction and small for gestational age status, 2005–2009 (N=8994)

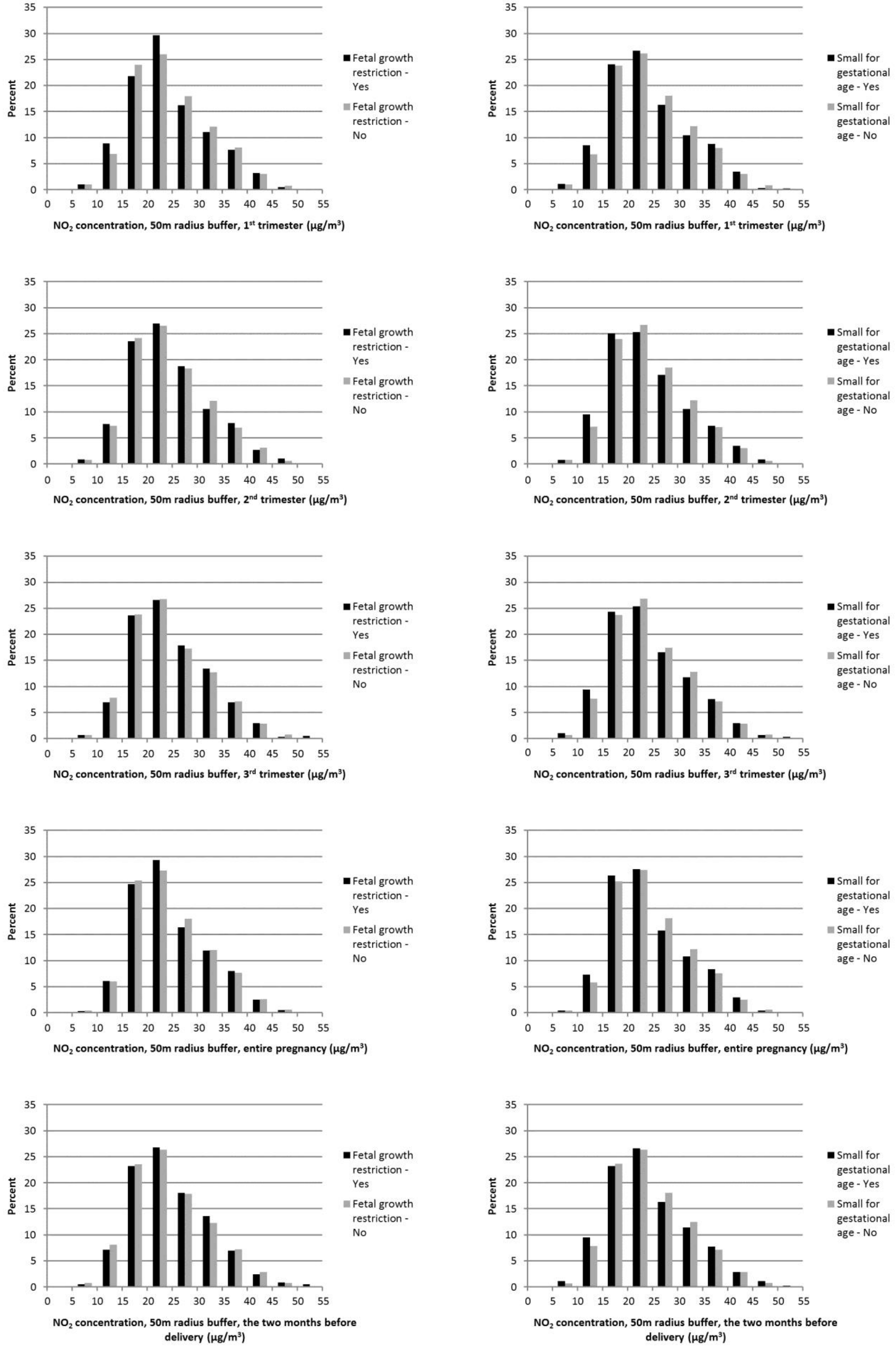

Figure S2. PM_{10} exposure, considering a 50m radius buffer, during different periods of pregnancy, according to fetal growth restriction and small for gestational age status, 2005–2009 (N=8994)

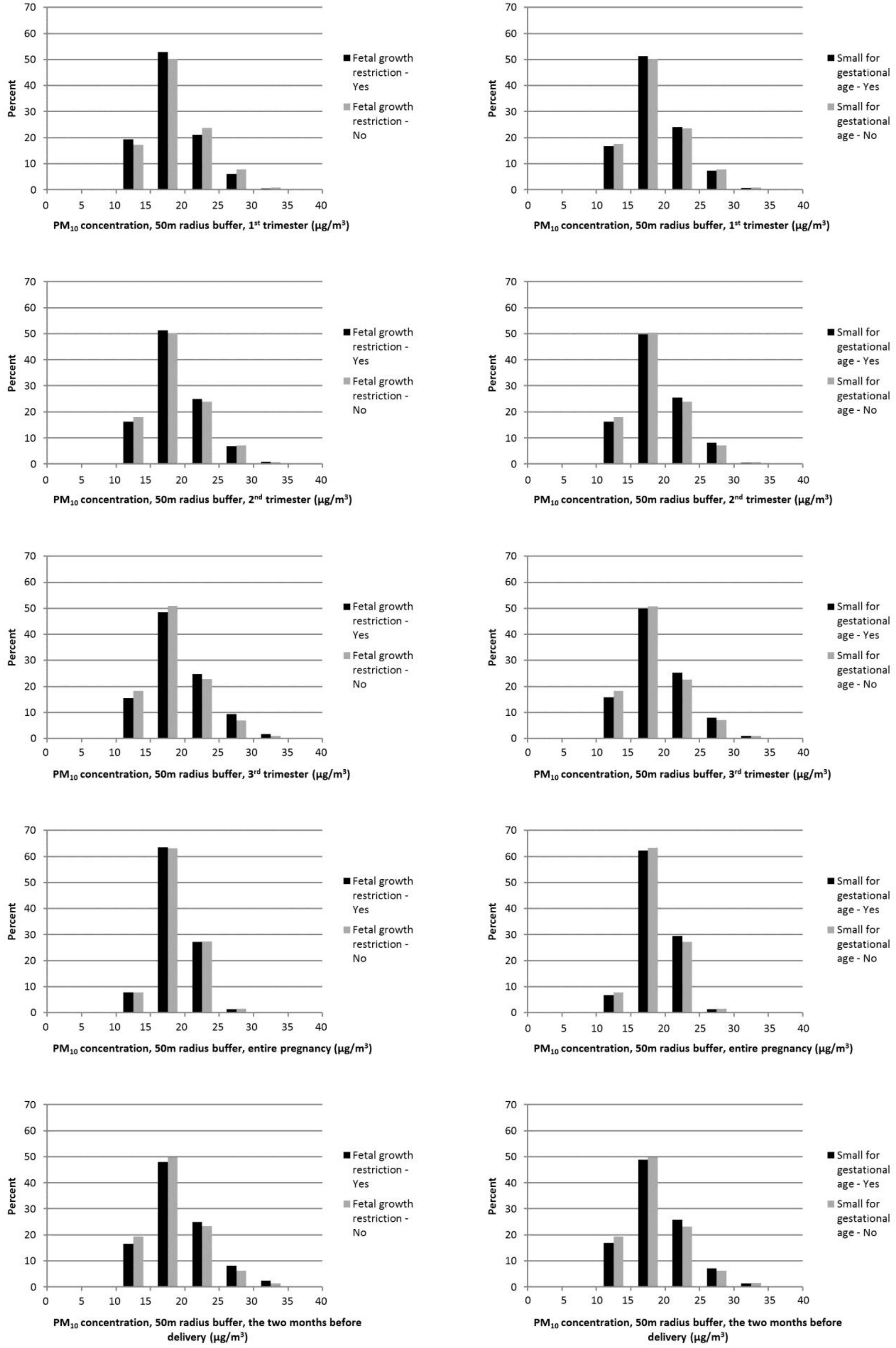

Figure S3. Total noise exposure, considering the average building noise levels in front of the entire façade (daily, day, evening, night equivalent, and day-evening-night A-weighted noise level), according to fetal growth restriction and small for gestational age status, 2005–2009 (N=8994)

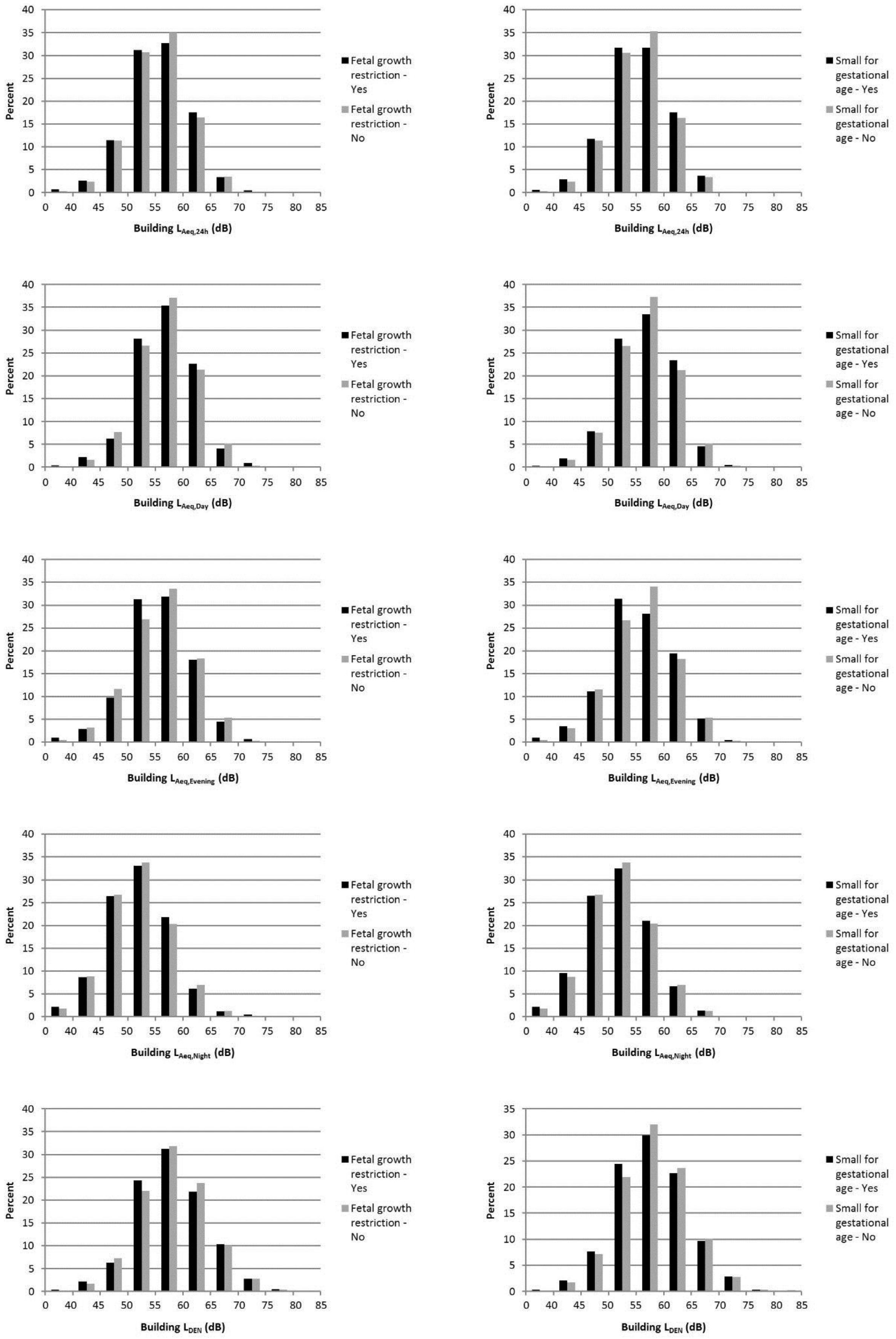

Figure S4. Representation of the Logit of the observed probability of fetal growth restriction or small for gestational age, according to total noise, NO_2 and PM_{10} exposure during the third trimester, 2005-2009 (N = 8994)

A) Logit of the observed probability of fetal growth restriction according to total noise

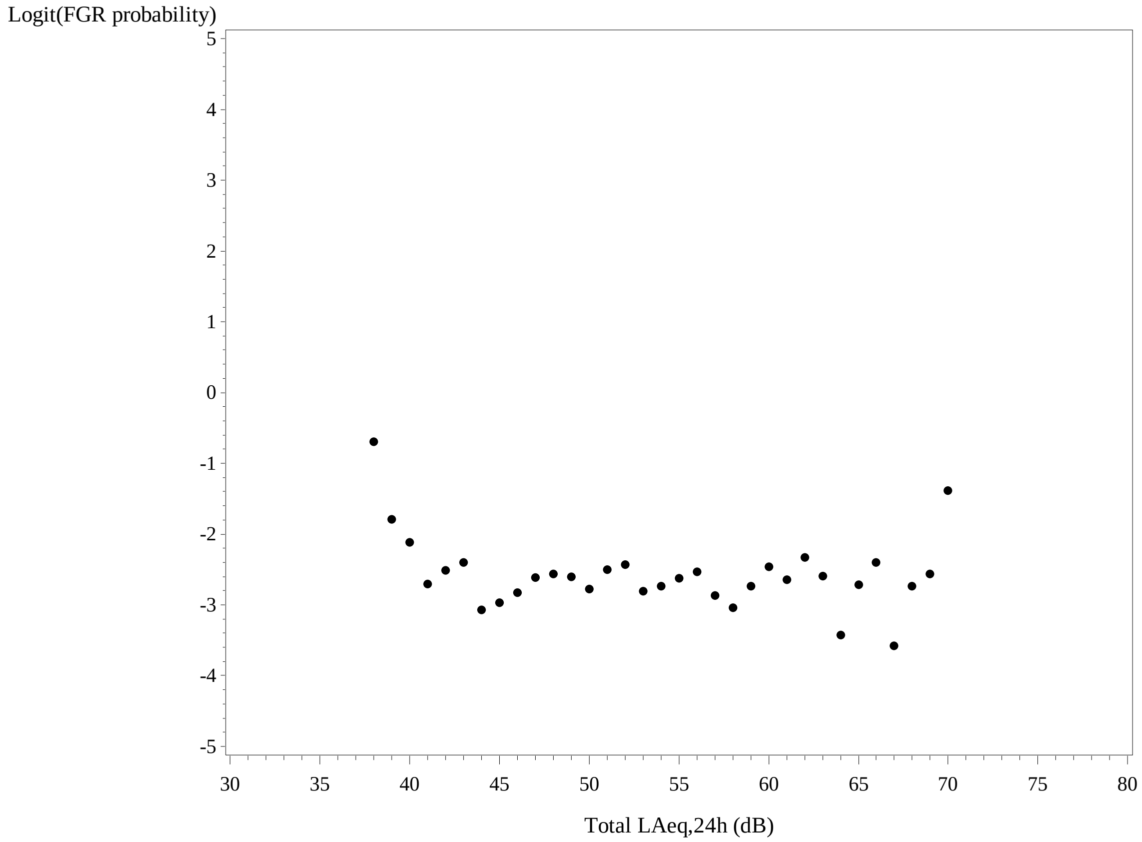

B) Logit of the observed probability of small for gestational age according to total noise

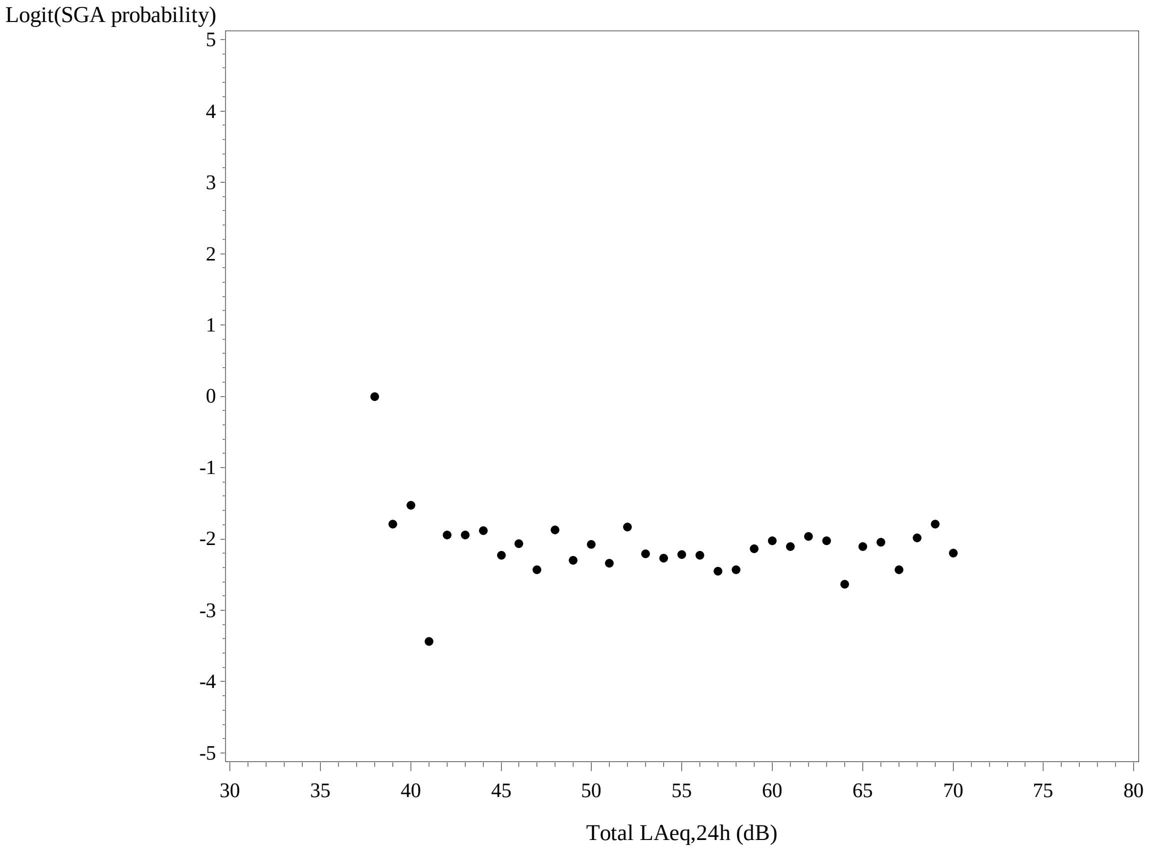

C) Logit of the observed probability of fetal growth restriction according to NO_2 exposure during the third trimester

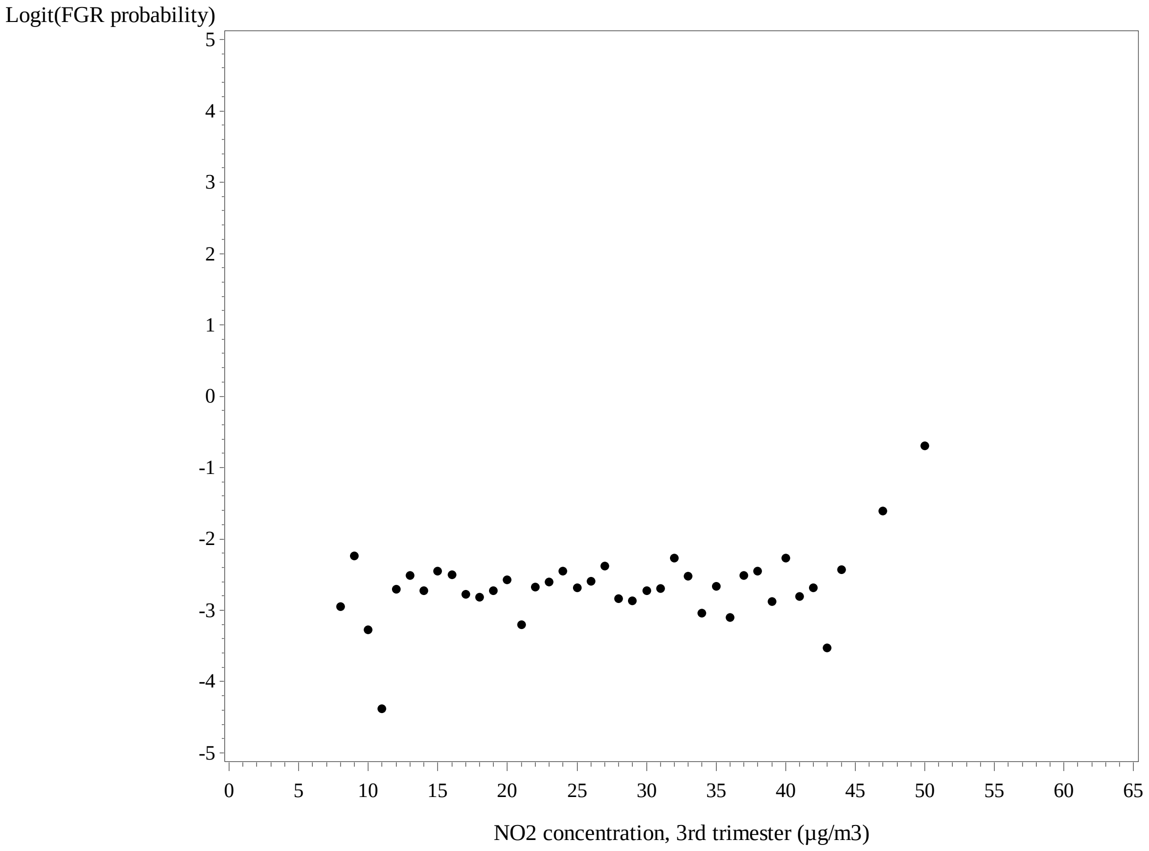

D) Logit of the observed probability of small for gestational age according to NO_2 exposure during the third trimester

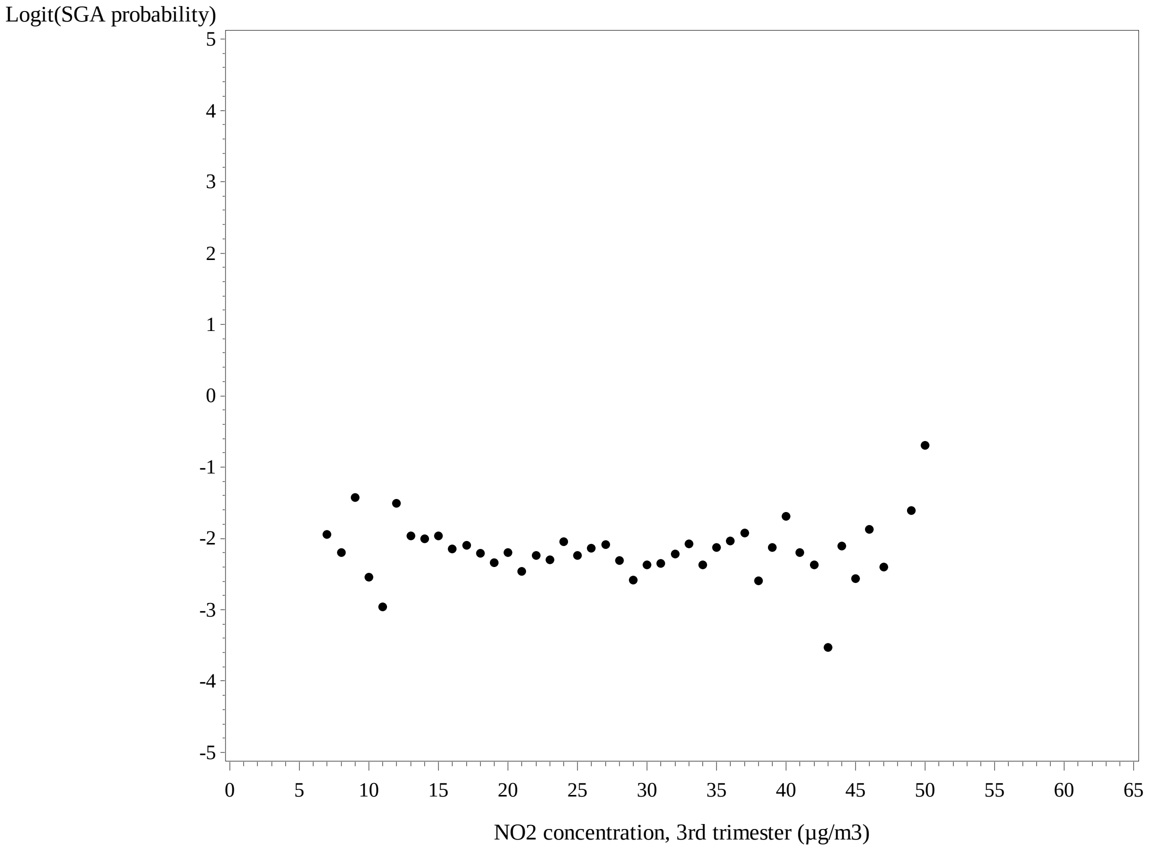

E) Logit of the observed probability of fetal growth restriction according to PM_{10} exposure during the third trimester

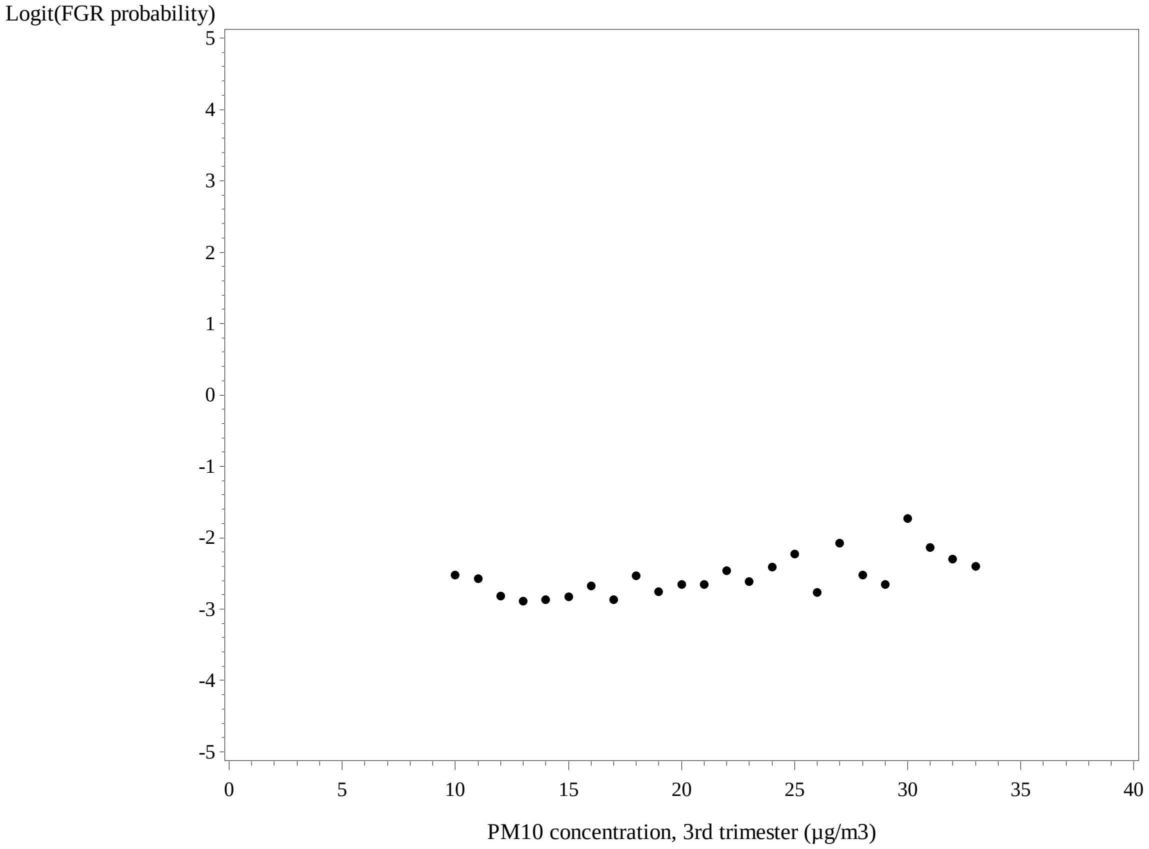

F) Logit of the observed probability of small for gestational age according to PM_{10} exposure during the third trimester

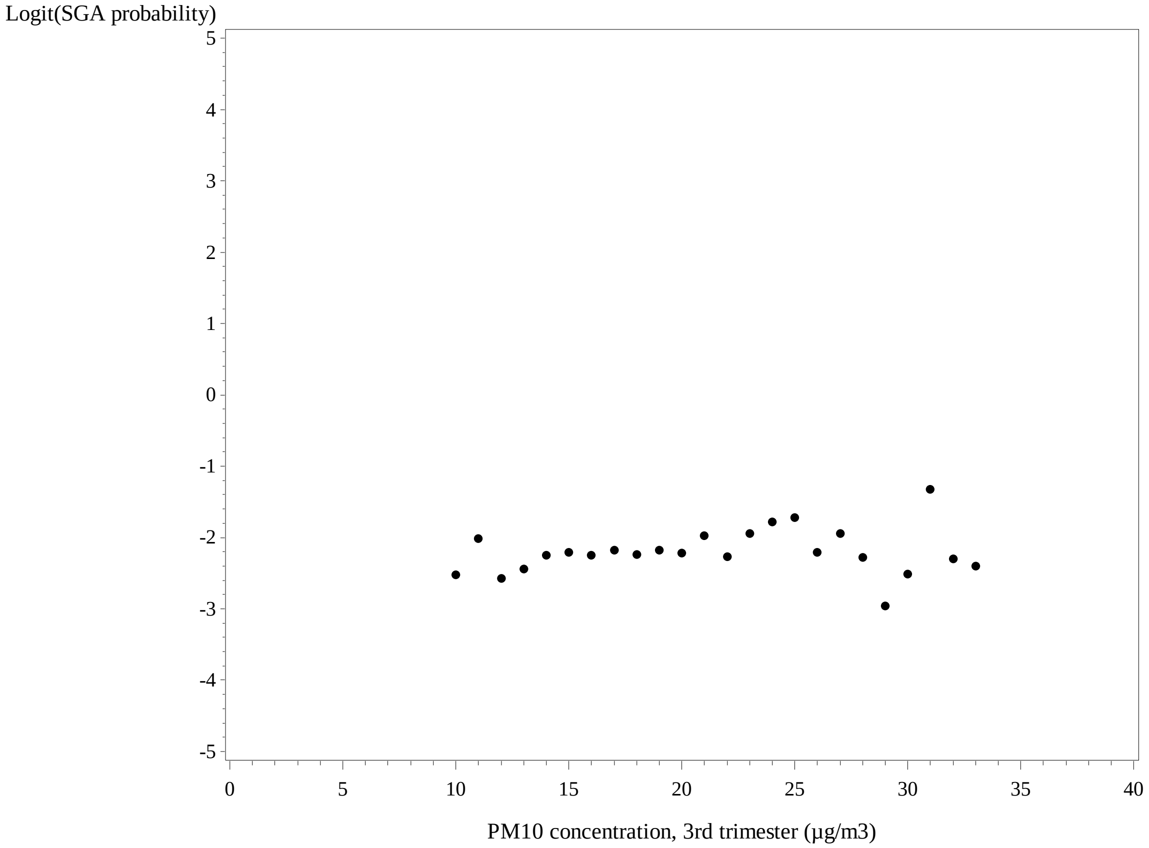

Supplement: Supplementary file 1 — Supplementary Information. [file 41598_2021_90788_MOESM1_ESM.pdf]
